# The antibody response to SARS-CoV-2 Beta underscores the antigenic distance to other variants

**DOI:** 10.1016/j.chom.2021.11.013

**Published:** 2022-01-12

**Authors:** Chang Liu, Daming Zhou, Rungtiwa Nutalai, Helen M.E. Duyvesteyn, Aekkachai Tuekprakhon, Helen M. Ginn, Wanwisa Dejnirattisai, Piyada Supasa, Alexander J. Mentzer, Beibei Wang, James Brett Case, Yuguang Zhao, Donal T. Skelly, Rita E. Chen, Sile Ann Johnson, Thomas G. Ritter, Chris Mason, Tariq Malik, Nigel Temperton, Neil G. Paterson, Mark A. Williams, David R. Hall, Daniel K. Clare, Andrew Howe, Philip J.R. Goulder, Elizabeth E. Fry, Michael S. Diamond, Juthathip Mongkolsapaya, Jingshan Ren, David I. Stuart, Gavin R. Screaton

**Affiliations:** 1 Wellcome Centre for Human Genetics, Nuffield Department of Medicine, University of Oxford, Oxford, UK; 2 Chinese Academy of Medical Science Oxford Institute, University of Oxford, Oxford, UK; 3 Division of Structural Biology, Nuffield Department of Medicine, University of Oxford, The Wellcome Centre for Human Genetics, Oxford, UK; 4 Diamond Light Source Ltd, Harwell Science & Innovation Campus, Didcot, UK; 5 Oxford University Hospitals NHS Foundation Trust, Oxford, UK; 6 Department of Medicine, Washington University School of Medicine, St. Louis, MO 63110, USA; 7 Peter Medawar Building for Pathogen Research, Nuffield Department of Medicine, University of Oxford, Oxford, UK; 8 Nuffield Department of Clinical Neurosciences, University of Oxford, Oxford, UK; 9 Department of Pathology and Immunology, Washington University School of Medicine, St. Louis, MO 63110, USA; 10 National Infection Service, Public Health England (PHE), Porton Down, Salisbury, UK; 11 Viral Pseudotype Unit, Medway School of Pharmacy, University of Kent and Greenwich, Chatham Maritime, Kent ME4 4TB, UK; 12 Department of Paediatrics, University of Oxford, Oxford, UK; 13 Department of Molecular Microbiology, Washington University School of Medicine, St. Louis, MO 63110, USA; 14 The Andrew M. and Jane M. Bursky Center for Human Immunology and Immunotherapy Programs, Washington University School of Medicine, St. Louis, MO 63110, USA; 15 Siriraj Center of Research Excellence in Dengue & Emerging Pathogens, Dean Office for Research, Faculty of Medicine Siriraj Hospital, Mahidol University, Bangkok, Thailand; 16 Instruct-ERIC, Oxford House, Parkway Court, John Smith Drive, Oxford, UK

**Keywords:** SARS-CoV-2, COVID-19, Beta variant, immune responses, spike protein, antibody, receptor-binding domain, structure, vaccine, neutralization

## Abstract

Alpha-B.1.1.7, Beta-B.1.351, Gamma-P.1, and Delta-B.1.617.2 variants of SARS-CoV-2 express multiple mutations in the spike protein (S). These may alter the antigenic structure of S, causing escape from natural or vaccine-induced immunity. Beta is particularly difficult to neutralize using serum induced by early pandemic SARS-CoV-2 strains and is most antigenically separated from Delta. To understand this, we generated 674 mAbs from Beta-infected individuals and performed a detailed structure-function analysis of the 27 most potent mAbs: one binding the spike N-terminal domain (NTD), the rest the receptor-binding domain (RBD). Two of these RBD-binding mAbs recognize a neutralizing epitope conserved between SARS-CoV-1 and -2, while 18 target mutated residues in Beta: K417N, E484K, and N501Y. There is a major response to N501Y, including a public IgVH4-39 sequence, with E484K and K417N also targeted. Recognition of these key residues underscores why serum from Beta cases poorly neutralizes early pandemic and Delta viruses.

## Highlights

• Generated 674 antibodies from patients infected with SARS-CoV-2 Beta variant
• 18 of 27 most potent mAbs target the 3 mutations in Beta RBD
• A major response to N501Y includes a public IgVH4-39 sequence
• Two antibodies recognize a neutralizing epitope conserved between SARS-CoV-1 and -2

## Graphical abstract

**Figure:**
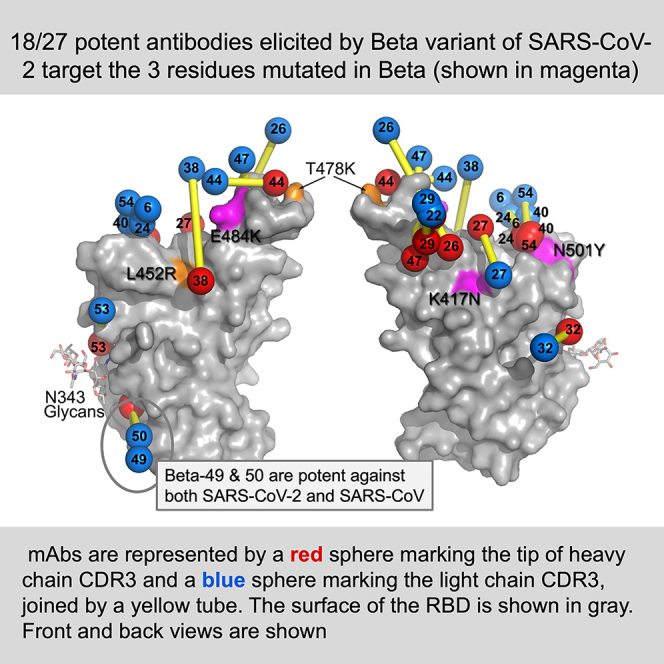

## Teaser

Liu et al. generated 674 antibodies from patients infected with the SARS-CoV-2 Beta variant. 18 out of 27 most potent neutralizing antibodies isolated target the 3 mutations present in the receptor-binding domain of this variant. This underscores the poor neutralization by Beta serum of early pandemic and Delta viruses.

## Introduction

Genomic surveillance of SARS-CoV-2 has identified many thousands of mutations in structural and non-structural proteins (COVID-19 Genomics UK (COG-UK) consortium, 2020). However, toward the end of 2020, viral variants were described that rapidly became the dominant strains locally and led to global spread and their designation as variants of concern (VoCs) Alpha, Beta, Gamma, and Delta. All of these variants contain multiple mutations in S and include changes in the receptor-binding domain (RBD), N-terminal domain (NTD), and in some cases the furin cleavage site between S1 and S2. The RBD mutations found in Alpha (N501Y), Beta (K417N, E484K, and N501Y), Gamma (K417T, E484K, and N501Y), and Delta (L452R and T478K) are located in or closely adjacent to the ACE2-interacting surface where they have the potential to modulate ACE2 interaction or disrupt the binding of neutralizing mAbs. Increased affinity for ACE2 has been demonstrated for Alpha, Beta, Gamma, and Delta (7-, 19-, 19-, and 2-fold, respectively) (Dejnirattisai et al., 2021b; Liu et al., 2021; Supasa et al., 2021; Zhou et al., 2021) and may play a role in increasing viral transmissibility.

A number of studies show vaccine efficacy against Beta to be reduced, and this corresponds with significantly reduced neutralization titers to Beta using serum obtained from early pandemic cases or vaccinees, when compared with neutralization of early pandemic strains (Madhi et al., 2021; Shinde et al., 2021; Zhou et al., 2021). The RBD mutations present in Beta (K417N, E484K, and N501Y) disrupt the binding of a number of potent neutralizing mAbs including some being developed for clinical use and likely, together with changes in the NTD, explain the antigenic distance between Beta and early SARS-CoV-2 strains (Zhou et al., 2021).

To understand the antigenic landscape of Beta, we produced a substantial panel of mAbs isolated from memory B cells of convalescent Beta cases. Of 674 mAbs generated from 5 donors 27 showed potent neutralizing activity (50% focus reduction neutralization assay [FRNT50] < 100 ng/mL). Neutralization assays against a variety of viral isolates indicated most mAbs showed restricted neutralization of a set of strains. A detailed structure-function analysis comprising 22 X-ray and cryo-EM structures is presented for 16 Fabs most in complexes with RBD or S, which allows an understanding of the changes in antigenicity of the Beta S protein. The majority of potent mAbs are directed to the mutations present in the Beta RBD, principally N501Y, and E484K, underscoring the small antigenic distance between Beta and Gamma, the larger distance between early pandemic strains and Beta, and interestingly, few anti-Beta mAbs can neutralize Delta, consistent with the extreme antigenic distance between Beta and Delta (Liu et al., 2021).

## Results

### Generation of mAbs from Beta-infected cases

**Figure 1:**
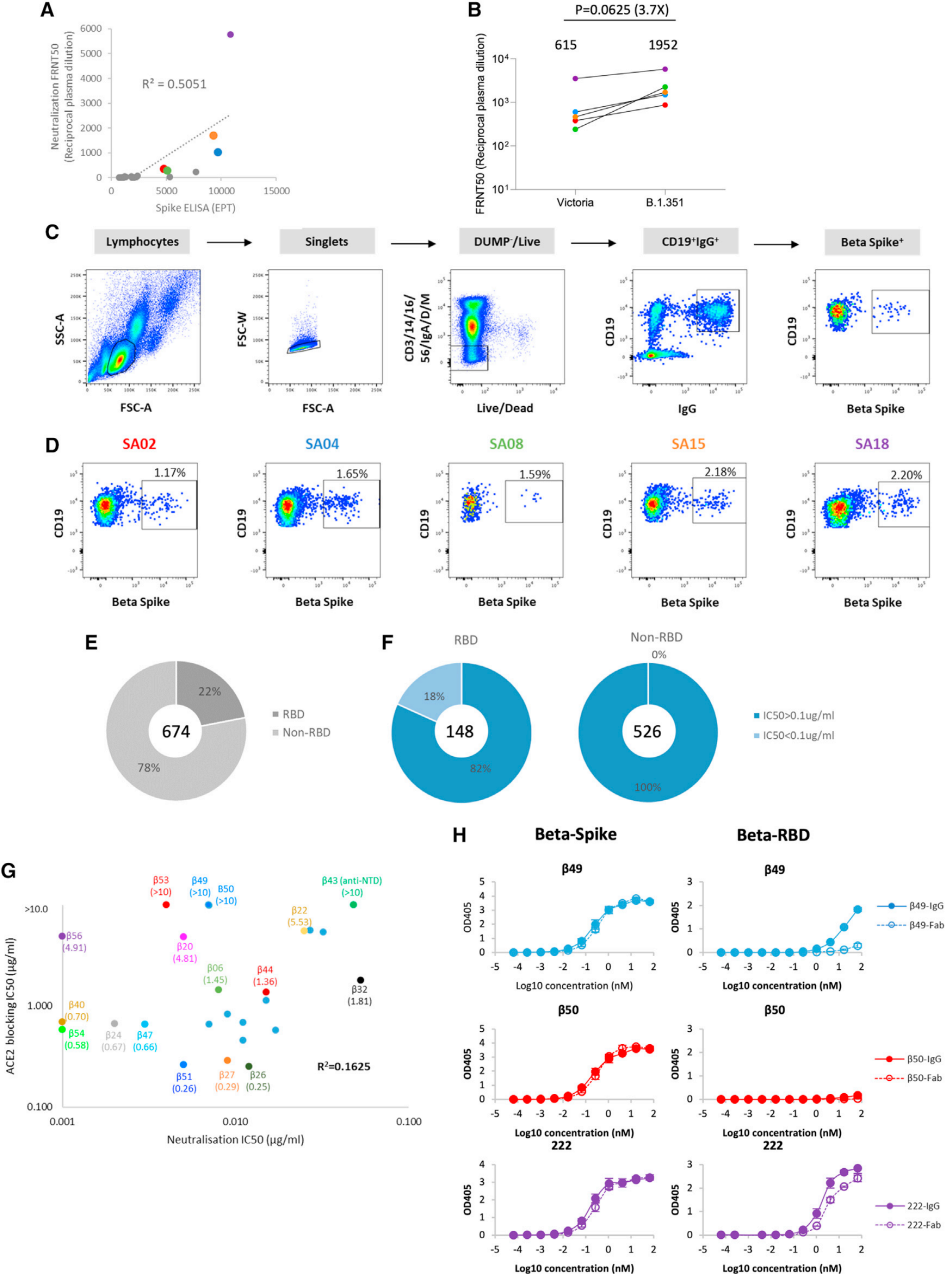
Isolation and characterization of Beta SARS-CoV-2-specific mAbs (A) Comparison of Beta SARS-CoV-2 neutralization and S binding ELISA by convalescent plasma from confirmed Beta SARS-CoV-2 infected donors. Plasma samples with FRNT50 >1:250 are highlighted and correspond to the cases shown in (D). (B) Neutralization titers against SARS-CoV-2 strain Victoria and the Beta variant for the 5 selected plasma samples with potent neutralizing properties, analysis used the Wilcoxon matched-pairs signed rank test and two-tailed p values were calculated; geometric means are indicated above each column. (C) Schematic of the Beta SARS-CoV-2 mAb isolation strategy. (D) Antigen-specific single B cells were isolated using labeled recombinant S protein as bait. The frequency of S-reactive IgG^+^ B cells was measured by FACS. (E) Epitope mapping of Beta SARS-CoV-2 specific mAbs against S and RBD were evaluated by ELISA. (F) Neutralization potencies (IC50) between anti-S (non-RBD) and anti-RBD mAbs against authentic Beta SARS-CoV-2 using a FRNT50 test. (G) Comparison of IC50 values for ACE2 binding and FRNT50 titers for the 27 potent mAbs, those selected for further structural study are highlighted. (H) Binding of Beta-49 and -50 Fab and IgG1 to Beta S trimer or Beta RBD measured by ELISA, comparison is made with binding of mAb 222, data are shown as mean ± SEM. See also Table S1A.

We collected plasma and peripheral blood mononuclear cells (PBMC) from 18 volunteers in the UK who had previously suffered infection with Beta as evidenced by viral sequencing or were inferred to have suffered Beta as they became infected after being isolated following contact with a proven Beta-infected case. Samples were taken 4–8 weeks following Beta infection, ELISA binding assays against full-length Beta S protein and FRNT assays were performed, and 5 cases with the highest titers were selected for further study (Figure 1A). For these selected cases, as expected, FRNT50 titers were higher for Beta than Victoria (an early Wuhan related viral isolate) (Figure 1B).

To isolate memory B cells, PBMC were stained with full-length double Strep tagged Beta S, and IgG^+^ B cells binding Beta S were single cell sorted (Figures 1C and 1D). IgVH and IgVL sequences were isolated by RT-PCR and full-length heavy chain (HC)- and light chain (LC)-expressing plasmids were created using a Gibson assembly reaction. Assembly products were transfected into HEK-293T cells in 96-well plates, and supernatants harvested and tested in neutralization assays against Beta virus at a final concentration of 0.1–1 μg/mL. Only those mAbs achieving >90% neutralization in this initial assay were selected for further study. In total, we obtained 674 Beta-specific mAb IgGs. Of these, 22% bound RBD epitopes, 78% bound to non-RBD epitopes, and 18% of the RBD-specific mAbs achieved >90% neutralization and were selected for further study (Figures 1E and 1F). Most were able to block ACE2 binding to S, although there were exceptions: mAb Beta-43, the single NTD-binding neutralizing antibody (Figure S1A), extremely potent RBD-binding mAb Beta-53 and potent but very weakly RBD-binding mAbs Beta-49 and -50 (Figures 1G and 1H).

### Cross reactivity of Beta-reactive mAbs

**Figure 2:**
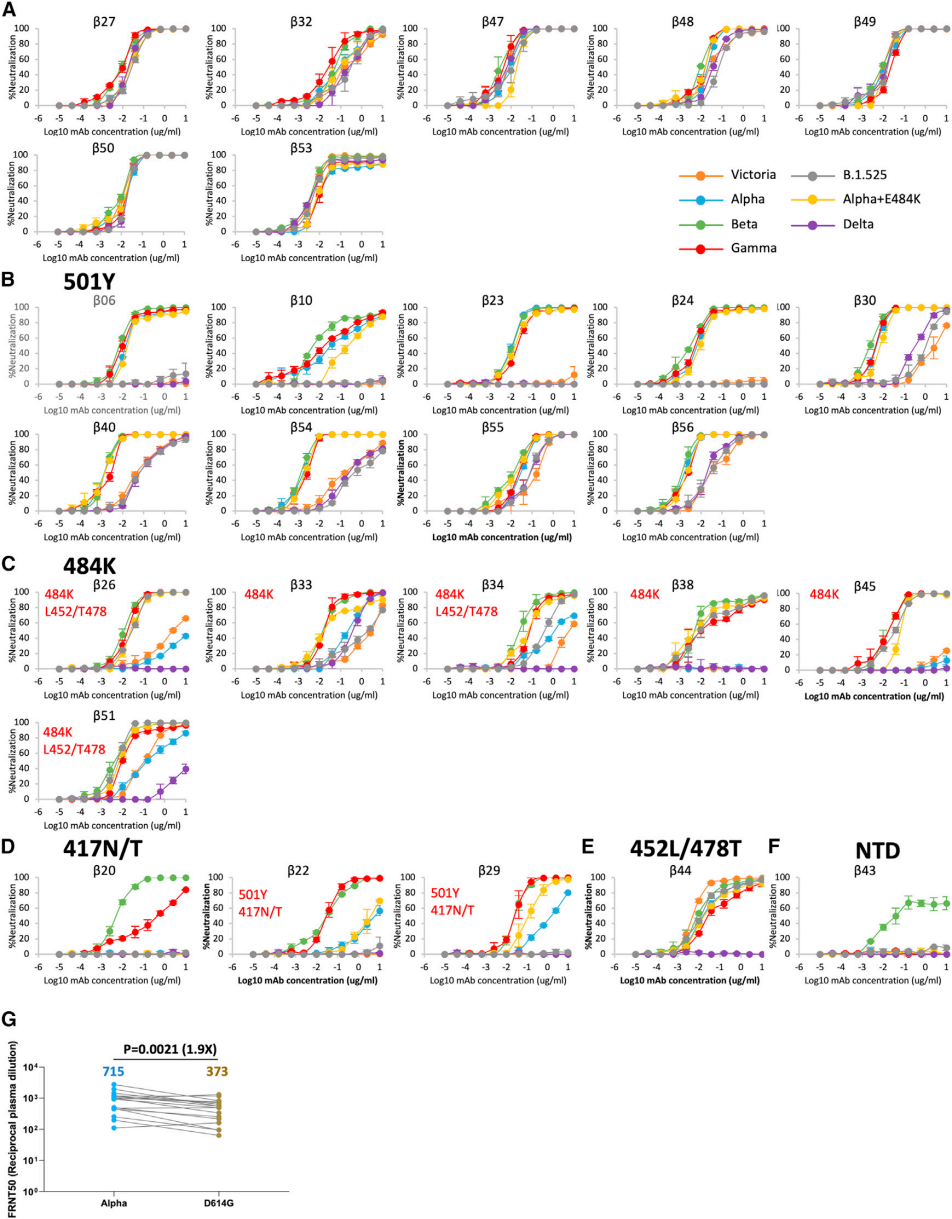
Cross reactivity of Beta-specific mAbs (A–F) Neutralization assays performed against Victoria, Alpha (N501Y), Beta (K417N, E484K, and N501Y), Gamma (K417T, E484K, and N501Y), Delta (L452R and T478K), Alpha+E484K (E484K and N501Y), and B.1.525 (E484K) live viral isolates with 27 potent Beta-specific mAbs. Titration curves are shown and mAbs grouped depending on the patterns of cross reactivity between the viral variants, potential binding determinants are indicated for the mAbs that show differential neutralization between isolates. Data are shown as mean ± SEM (A) Fully cross-reactive mAbs, (B) N501Y-dependent mAbs, (C) E484K-dependent mAbs, (D) K417N/T-dependent mAbs, (E) L452R/T478K-dependent mAbs, and (F) a single NTD-binding mAb. FRNT50 values are reported in Table S1. (G) FRNT50 titers of 17 Alpha convalescent sera against Alpha and B.1 (D614G), analysis used the Wilcoxon matched-pairs signed rank sum test, and two-tailed p values were calculated; geometric means are indicated above each column. See also Figure S1.

We performed live virus neutralization assays using the following viruses, containing the indicated changes in the RBD: Victoria (an early Wuhan related strain), Alpha (N501Y), Beta (K417N, E484K, and N501Y), Gamma (K417T, E484K, and N501Y), Delta (L452R and T478K), Alpha+E484K (E484K and N501Y), and B.1.525 (E484K) (Figures 2A–2F; Table S1A).

Many mAbs showed extremely potent neutralization of Beta, with FRNT50 down to 1 ng/mL (Table S1A). Cross reactivity between the different viral variants was mixed, some mAbs such as Beta-27, -32, -47, -48, -49, -50, and -53 showed full cross reactivity with <10-fold difference between FRNT50s (Figure 2A). A large group of mAbs (Beta-6, -10, -23, -24, -30, -40, -54, -55, -56) showed good neutralization of Alpha, Beta, Gamma, and Alpha+ viruses, with either reduced or completely absent neutralization of Victoria, B.1.525 (E484K), and Delta viruses (Figure 2B). Alpha, Beta, and Gamma have a single mutation in common, N501Y, and we propose that the presence of the N501Y mutation creates an epitope for RBD recognition in Beta infection.

The E484K mutation disrupts the binding of many potent mAbs generated from cases infected with early pandemic viruses, and we expected Lys-484 to be recognized by Beta-neutralizing mAbs (Zhou et al., 2021). Six mAbs show evidence of Lys-484 interaction (Beta-26, -33, -34, -38, -45, and -51), with reduced activity to Alpha, but regaining activity on Alpha+484K (Figure 2C). Three mAbs, Beta-20, -22, and -29 showed maximum activity toward Beta and Gamma, suggesting that they recognize an epitope related to the K417N/T changes in Beta and Gamma, respectively. mAbs Beta-22 and -29 showed some neutralization of Alpha and Alpha+484K suggesting that they recognize an epitope comprised of Asn/Thr 417 + Tyr-501 (Figure 2D). Four mAbs (Beta-26, -34, -44, and -51) showed selective loss of neutralization to Delta (FRNT50 > 10μg/mL). We propose Beta-44 is sensitive to L452R/T478K mutations while Beta-26, -34, and -51 recognize an epitope composed of Glu-484 + Leu-452/Thr-478 (Figures 2C and 2E). Finally, the single potent NTD-binding mAb Beta-43 was completely specific for Beta (Figures 2F and S1A).

We have previously looked at the neutralization of a panel of VoCs using serum collected from cases infected with Alpha (Supasa et al., 2021) and found that following Alpha infection, the response formed is quite cross-reactive between variant viruses. Paradoxically, we found that Alpha serum neutralized Victoria as efficiently as Alpha, which would not be expected if the response to Tyr501 in Alpha infection was similar to that seen in Beta infection. However, Alpha also contains the mutation D614G, which is not found in Victoria/Wuhan, we therefore went back and tested neutralization of a version of the early pandemic virus (B.1) containing the additional D614G mutation, using 17 Alpha sera. The neutralization titer of B.1 by Alpha serum was reduced 1.8-fold compared with the neutralization titer of Alpha using Alpha serum (p = 0.0208) (Figure 2G), consistent with some Tyr501 responses in Alpha serum.

### Antibody gene usage

**Figure 3:**
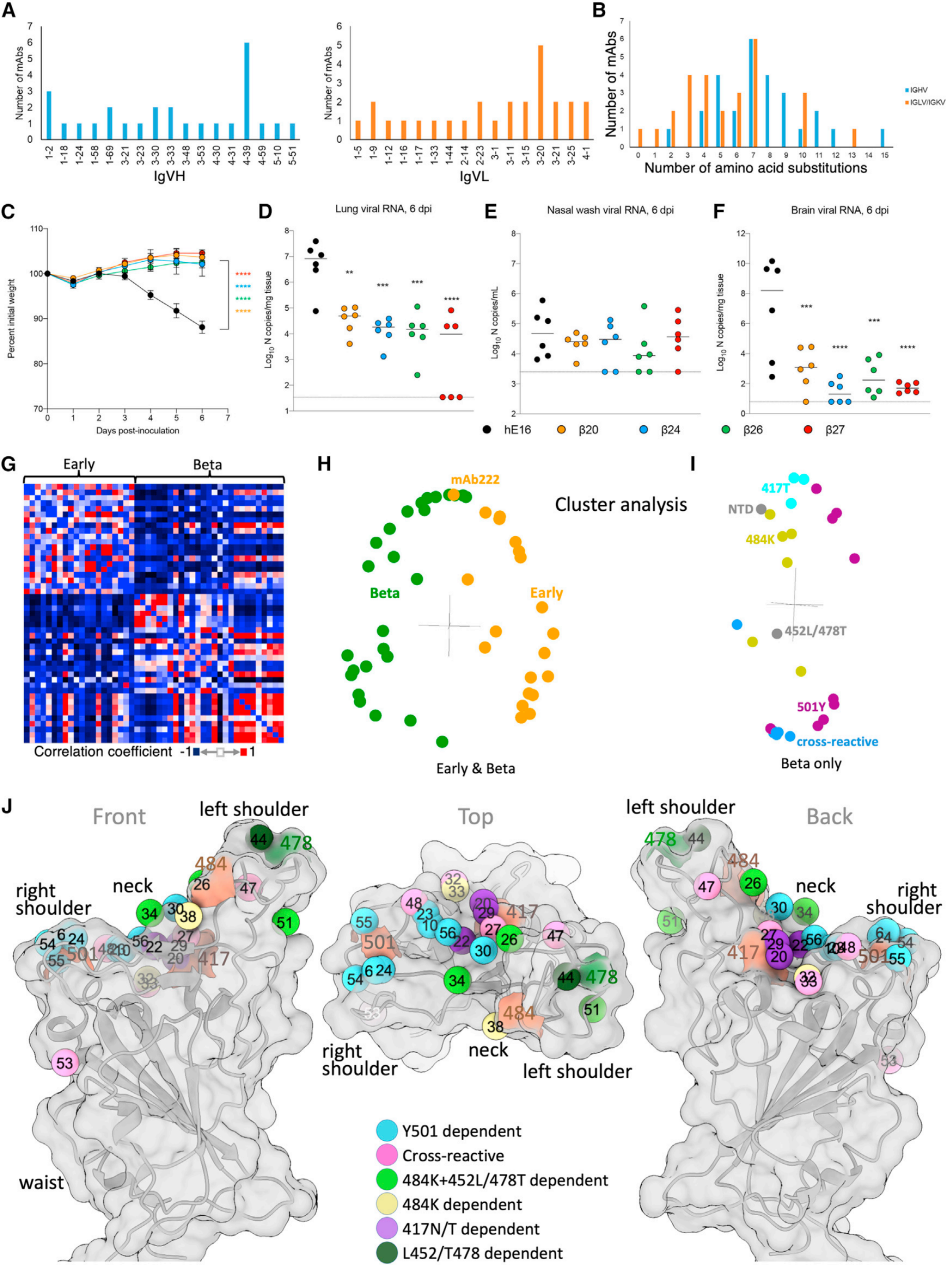
Gene usage, therapeutic use in K18-hACE2 mice, and computational analysis of responses (A) IgVH and IgVL gene usage for the 27 potent mAbs. (B) Amino acid substitutions in IgVH and IgVL for the 27 potent mAbs. (C–F) 8-week-old female K18-hACE2 transgenic mice were administered 10^3^ FFU of SARS-CoV-2 Beta strain by intranasal inoculation. One day later, mice received a single 10 mg/kg dose of the indicated mAb treatment by intraperitoneal injection. Tissues were collected at 6 dpi. (C) Weight change following infection with SARS-CoV-2 (mean ± SEM; n = 6 mice per group, two experiments; one-way ANOVA with Dunnett’s test of area under the curve: ^∗∗∗∗^p < 0.0001). Viral RNA levels in the lung (D), nasal wash (E), and brain (F) (line indicates median; n = 6 mice per group, two experiments; one-way ANOVA with Dunnett’s test with comparison to control mAb: ^∗∗^p < 0.01, ^∗∗∗^p = 0.001, ^∗∗∗∗^p < 0.0001. Dotted line indicates the limit of detection of the assay. (G) Cross-correlation matrix showing agreement of neutralization titers for mAbs against seven variants of SARS-CoV-2. Every antibody is associated with a vector containing the residual neutralization titer after subtracting the mean for each variant and normalizing to a standard deviation of 1. (H–J) Each point (I and J) in the matrix is colored according to the dot product between vectors for antibody (I and J). (H) Major modes of variation after singular value decomposition of the matrix in (G). (I) Major modes of variation after singular value decomposition of a matrix similar to (G) but calculated for Beta mAbs and colored according to their designation as a fully cross-reactive, 501Y-specific, 484K-specific, or 417T-specific antibody. (J) Mapping of the Beta mAbs based on BLI competition measurements (STAR Methods; Dejnirattisai et al., 2021a). The mean positions of the mAbs are shown as spheres. Numbers match the antibody definitions in Tables S1A and S1B (βs omitted for clarity), colored by one aspect of the serological properties e.g., Y501-dependent indicates potent neutralization is only observed for those viruses with Tyr-501. Anatomical terms relate to the torso analogy (Dejnirattisai et al., 2021a). The RBD is shown as a semi-transparent surface with cartoon embedded. The outer two are related by 180° rotation about the vertical axis, and the central view is related to the “front” view by a 90° rotation about the horizonal axis. See also Figure S2 and Table S1B.

IgVH and IgVL gene usages for the 27 potent RBD-binding, Beta-reactive mAbs are shown in Figure 3A with the individual mAb and patients from whom they were isolated shown in Table S1B. The 7 fully cross-reactive mAbs came from diverse IgVH families, apart from Beta-49 and -50, which were IgVH1-69. Beta-27 is from the IgVH3-53 gene family, which generates a public response to RBD, highly represented in repertoires isolated from individuals infected with early pandemic strains (5/20 potent mAbs FRNT50 < 100 ng/mL in our previous studies [Dejnirattisai et al., 2021a]), but was only represented once in this set. Likewise, Beta-47 belongs to a public gene family, IgVH1-58, found in a number of potent mAbs isolated from early pandemic infections (4/20 in our previous studies).

Tyr-501-reactive mAbs were the most represented, with 11/27 examples. In 9 of these, Tyr-501 was dominant, and in a further 2 (Beta-22 and -29), Tyr-501 played a role in addition to Asn-417. 6/11 of the Tyr-501-reactive mAbs used IgVH 4-39 (Beta-6, -10, -23, -40, -54, and -55), making IgVH4-39 a public antibody response following Beta infection, partly explaining the repositioning of the response toward Tyr501 relative to early pandemic strains (Yuan et al., 2020a). In our antibody panel generated from early pandemic cases 0/20 potent mAbs were IgVH4-39 compared with 6/27 in the Beta set (p = 0.0241, two-proportion Z test). The six Lys-484-reactive mAbs came from diverse IgVH backgrounds, whereas 2/3 of the Asn/Thr-417 + Tyr-501-reactive mAbs were VH3-30.

The 27 Beta-reactive mAbs showed relatively low levels of somatic mutations with median 7 changes in IgVH and IgVL (Figure 3B), which is consistent with the low level of hypermutation seen when analyzing mAbs following infection with early pandemic strains (median IgVH = 5 and IgVL = 3) (Dejnirattisai et al., 2021a; Rogers et al., 2020).

In summary, potent mAbs derived from Beta-infected cases differ considerably in their cross reactivity between variant viruses compared with mAbs isolated from early convalescent cases. The Tyr-501 and Lys-484 epitopes dominate the response leading to failure of a number of mAbs to neutralize Victoria and Delta, which underscores the antigenic distance between these viruses and Beta (Liu et al., 2021). Neutralization of Delta is further impaired by a subset of mAbs that are sensitive to the RBD mutations in Delta, explaining why Beta and Delta (and Gamma/Delta) occupy the most distant positions on an antigenic map (Liu et al., 2021).

### Potent mAbs protect against Beta infection in mice

To test the activity of mAbs elicited against Beta *in vivo*, we utilized a human ACE2 transgenic mouse model (McCray et al., 2007; Winkler et al., 2020). Four representative mAbs from different epitope classes were selected: Beta-20, which recognizes the K417N/T mutation and can potently neutralize Beta and to a lesser extent Gamma, Beta-24, which is specific to the N501Y mutation present in Alpha, Beta, and Gamma, Beta-26, which recognizes the E484K mutation found in Beta and Gamma, and Beta-27, the IgVH3-53 fully cross-reactive mAb, which neutralizes all variants similarly.

Mice were inoculated with 10^3^ FFU of Beta and at 24 h post-inoculation, were administered a single 10 mg/kg dose of mAb via intraperitoneal injection. All four Beta-elicited mAbs, but not an isotype control mAb (hE16), prevented weight loss over the 6 days following inoculation and reduced viral loads in the lung and brain but not in nasal washes (Figures 3C–3F). These results demonstrate that each of the mAbs we tested can efficiently reduce the severity of infection and prevent systemic disease but do not prevent viral infection in the upper respiratory tract.

### Quantitative dissection of similarities and differences in mAb responses

The 27 potent RBD-binding Beta mAbs appeared markedly different compared with the 20 potent mAbs we generated following infection with early pandemic strains. To quantify this, we devised a neutralization-correlation method, comparing the neutralization results for mAbs against seven virus strains between all possible pairs of the 47 potent mAbs. The metric for the comparison was the correlation coefficient between the neutralization results for the two mAbs (see STAR Methods). Displaying this as a heat matrix (Figure 3G) reveals clear differences between the Beta and early pandemic mAbs. Cluster analysis effectively separated the two sets (Figure 3H), demonstrating that the pattern of strain neutralization is similar within but different between the two sets. This segregation is highly significant (p < 0.00001 for the Mann-Whitney U test) confirming distinct patterns of strain neutralization specific to the eliciting virus. Further cluster dissection of the Beta mAbs (Figure 3I) segregates them according to their specificities to the individual RBD mutations described in Figures 2A–2F.

### Mapping of mAbs binding using BLI

We have previously built a high-resolution 3D map, consistent with X-ray structure determination, of early pandemic mAbs binding to the RBD using biolayer interferometry (BLI) (Dejnirattisai et al., 2021a). We therefore acquired a matrix of pairwise BLI measurements on 23 of the 26 most potent RBD-binding Beta mAbs and some pre-pandemic mAbs of known binding position. Combining the BLI data with precise structural information provided reliable mapping information on their positioning on the RBD (prediction for 7 mAbs Beta-6, -22, -24, -27, -44, -53, and -54 were in error by on average 2 Å). The mAbs do not segregate into epitopes, rather cluster analysis (STAR Methods) shows that they nearly all fall in an arc (Figure S2), spanning the neck and shoulders of the RBD (Dejnirattisai et al., 2021a) (Figure 3J).

There is an excellent correlation with the critical residue assignments made above (Figure 3J). For example, Beta-44, sensitive to L452R/T478K mutations, is perched adjacent to residue 478, while mAbs, Beta-20, -22, and -29 suggested to recognize an epitope related to residue 417 are tightly clustered atop this residue. Interestingly, mAbs Beta-49 and -50 showed very low affinity to Beta RBD, although they bound tightly to full-length S (Figure 1H) and hence could not be mapped.

### Structures of anti-Beta Fab/RBD complexes

**Figure 4:**
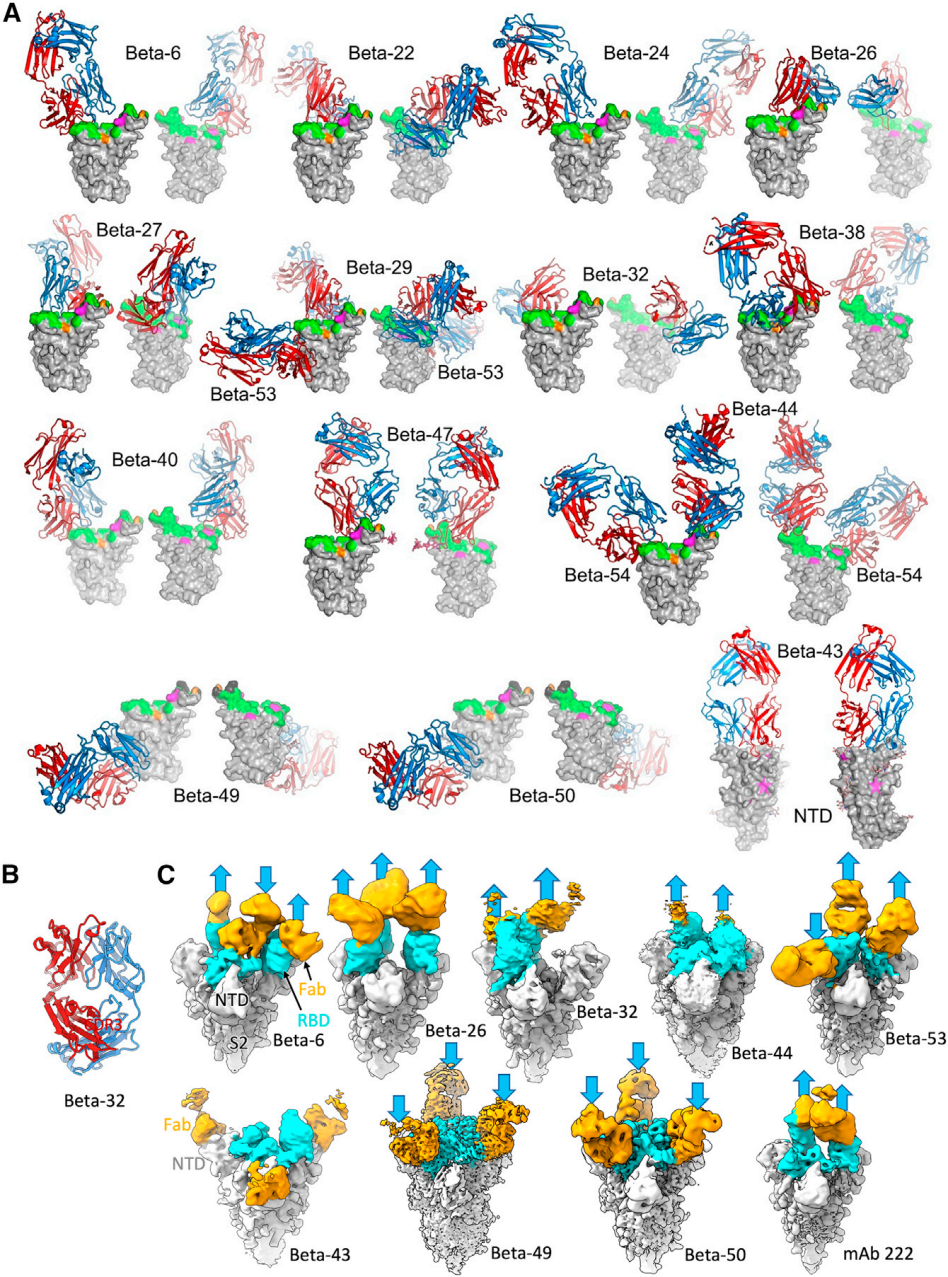
Overall structures of Beta RBD/Beta S complexes with Beta mAb Fabs reported in this paper (A) Front and back views of Beta RBD/Beta Fab complexes. Fabs drawn as ribbons with HC red and LC blue, and RBDs as gray surfaces with ACE2 footprint in green, mutation sites of the Beta variant in magenta and Delta variant in orange. All structures were crystallographic except Beta-26 and -32, which were derived by cryo-EM. (B) Crystal structure of Beta-32 Fab with HC red and LC blue. (C) Cryo-EM maps of Beta S complexes with Beta-6, -26, -32, -44, -53, -43, -49, and -50 and early pandemic mAb-222 Fabs. The bound Fabs are orange, RBD domains cyan, and the rest of S gray. Arrows indicate the RBD orientations. See also Figure S3 and Tables S2 and S4.

Structural analyses were performed for representative potent anti-Beta mAbs, selected for their serological properties. Crystal structures were determined for complexes of RBD with 15 different Beta Fabs: 6, 22, 24, 27, 29, 38, 40, 44, 47, 49, 50, 53, and 54, the Beta-43/NTD complex (Figure 4A), and also for Fab Beta-32 alone (Figure 4B), at resolutions between 1.7 and 4.0 Å (STAR Methods; Table S2). In addition, cryo-EM structures were obtained for eight Beta S complexes, with Fabs of Beta-6, -26, -32, -43, -44, -49, -50, and -53, plus Beta S complexed with Fab of cross-reactive mAb-222 identified earlier (Dejnirattisai et al., 2021b) (Figures 4C and S4; STAR Methods).

### Structural definition of IgVH4-39 public mAbs against the Tyr-501 RBD

**Figure 5:**
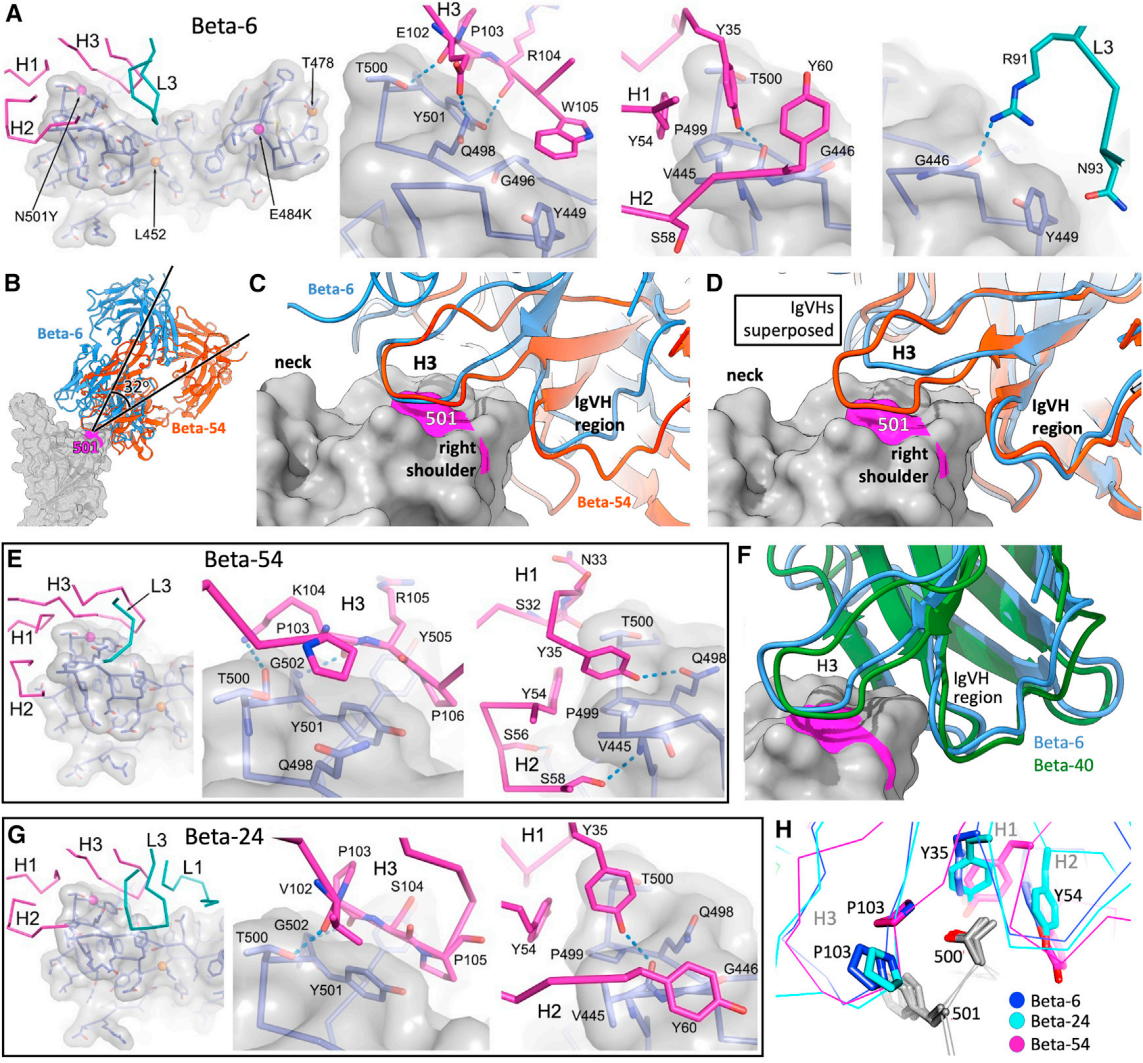
Structural details of IgVH4-30 and IgVH4-39 Beta Fab complexes (A) Beta-6, Beta-RBD interactions. Left panel shows interacting CDRs (HC magenta, LC cyan) with the Beta-RBD (semi-transparent gray surface, side chains as blue sticks, mutation sites of Beta [magenta] and Delta [orange] variants shown as spheres). Interactions of H3, H2, H1, and L3 loops are shown in the adjacent panels. (B) Comparison of binding orientations for Beta-6 (blue) and Beta-54 (red). (C) Closeup of (B) showing engagement of CDR-H3s with Tyr-501 (magenta). (D) Same as (C) but IgVHs are overlapped instead of RBDs. (E) Interactions of Beta-54 with Beta RBD. (F) Comparison of binding modes of Beta-40 IgVH (green) and Beta-6 (Blue). (G) Interactions of Beta-24 with Beta RBD. (H) Common features of the engagement used by Beta-6 (blue), -24 (cyan), and -54 (magenta). Y35 of CDR-H1 and Y54 of CDR-H2 are conserved among the IgVH4-30 and IgVH4-39 Beta mAbs reported here. See also Tables S2 and S3.

We determined structures for Beta-6 and Beta-54 of the widely used IgVH4-39 gene family. Both interact strongly with Tyr-501. Beta-6 perches atop the right shoulder of the RBD (Figure 5A), with principal contacts contributed by the HC (520 Å^2^), while the LC makes very few contacts (124 Å^2^), limited to LC CDR3 (L3) (Figure 5A). The interaction area is relatively small, with interactions heavily focused around residue Tyr-501 (Figure 5A) with the three HC CDRs (H1-3) wrapping around the right shoulder. In particular, the hydroxyl group of Tyr-501 makes a hydrogen bond to the H3 main chain (Figure 5A).

Beta-54 has a significantly different angle of attack on the RBD compared with Beta-6 (Figure 5B), with the Fab pivoting by 32° around residue 501 on the RBD. This change in binding pose appears to derive from the difference in length of the H3 (15 residues in Beta-6, 18 in Beta-54). Altering the pose allows the different length H3 loops to make similar contacts (Figure 5C), while the H1 and H2 contact regions pivot round. Superposition of the IgVH portions (Figure 5D) shows that, with the exception of H3, the HC variable domains are very similar, although details of the interaction with the RBD differ (Figures 5A and 5E). Residues Tyr-35 (H1) and Tyr-54 (H2) are central in allowing the pivoting and characteristic of the IgVH4-39 gene family (they are also found in some other IgVH families and insufficient to convey this binding mode alone, Figure 5E; Table S3). Overall, the HC interaction area is similar for both mAbs (461 and 54 Å^2^ for the HC and LC, respectively, of Beta-54).

Beta-6 and -54 are examples of the two classes of IgVH4-39 Beta mAbs, with the shorter loop Beta-6-like mAbs forming the major class (Table S3). We suggest that the two distinct classes of H3 are accommodated by some degeneracy in the H1 and H2 interactions although related interactions in the IgVH4-39 portions are maintained (considering all interacting residues of both antigen and antibody, 22 are common between the two mAbs, out of 28 in Beta-6 and 34 in Beta-54).

As a test of the proposal that for IgVH4-39 mAbs H3 length modulates binding pose, a further low-resolution X-ray structure was determined for Beta-40 Fab bound to Beta RBD. Beta-40 has the canonical 15 residue H3 found in four of the IgVH4-39 mAbs, including Beta-6. As predicted, the angle of attack of Beta-40 is essentially identical to Beta-6 and H1H2 positioning is very similar (Figure 5F). The model is further supported by the structure of the Beta RBD/Beta-24 complex. Beta-24 belongs to the closely related IgVH4-30 gene family with H1 and H2 regions very similar to IgVH4-39 (Table S3) and a canonical H3 length of 15 residues. Beta-24 has a mode of engagement essentially identical to Beta-6 (Figures 5G and 5H), despite having a markedly different sequence within the 15-residue H3 (Table S3). While the interactions in part recapitulate those of Beta-6 there are also differences, the LC interaction area is more than doubled for Beta-24 (to 284 Å^2^), while the HC contacts are slightly reduced (458 Å^2^). Nevertheless, some common HC interactions define the similar mode of association (Figure 5H). Thus, the hydroxyl group of Tyr-35 forms a hydrogen bond with the RBD carbonyl oxygen of Val-445 and the carbonyl of residue Val-102 interacts with the RBD hydroxyl of Thr-500. However, the interactions with Tyr-501 differ, with a hydrogen bond formed in Beta-6, compared with hydrophobic stacking interactions in Beta-24. Interestingly, the LC interactions are not homologous—in both mAbs a hydrogen bond is formed to the carbonyl of Gly-446; however, in Beta-6 this is contributed by the guanidinium group of Arg-91 (Figure 5A), while in Beta-24 the hydrogen donor is the hydroxyl group of Tyr-91.

To investigate IgVH4-39 antibody binding in the context of the complete S trimer, the Beta S/Beta-6 Fab complex structure was determined by cryo-EM, which showed attachment in a standard way with 2 RBDs in the up configuration; however, all three RBDs bear bound Fabs (Figure 4C). In summary, the recurrent use of IgVH4-39 appears to signal a frequent public binding mode targeting residue Tyr-501, despite the mAbs harboring a wide range of different IgVL gene families (Table S1A).

### IgVH1-69 mAbs target a neutralizing epitope conserved between SARS-CoV-1 and -2

**Figure 6:**
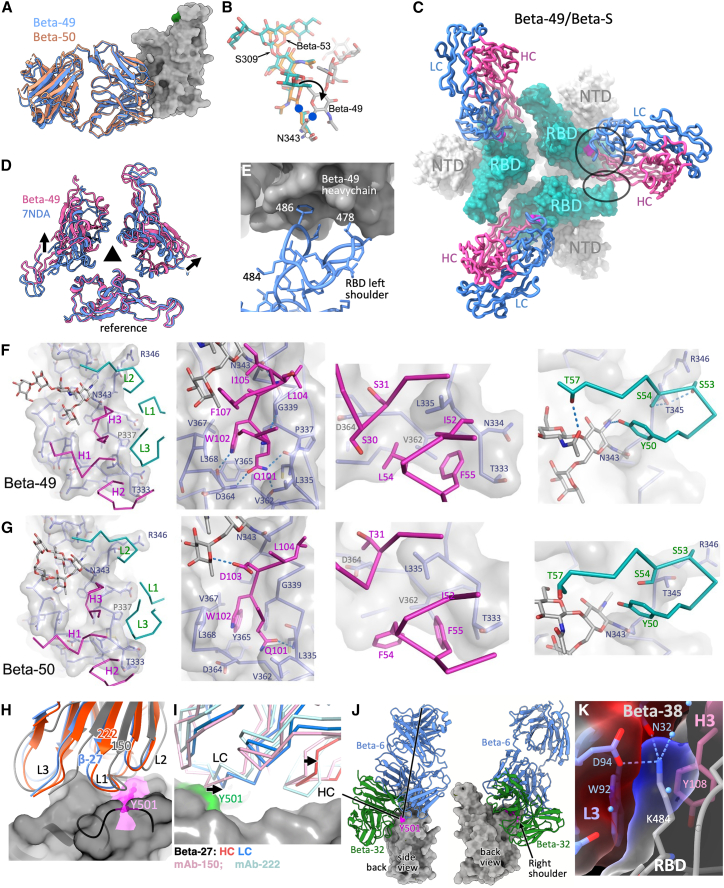
Engagement of other Beta IgVH Fabs with the Beta RBD (A) Almost identical binding of Beta-49 (blue) and Beta-50 (salmon) to the RBD. (B) Overlay of N343 RBD glycan from the (green) (Pinto et al., 2020), Beta-53 (yellow) and Beta-49 (gray) complexes, the side chain rotated into an unfavorable conformation in the latter. (C) Top view of the Beta-49 Fab/Beta S complex. S is shown as a surface (RBD cyan, position of glycan attachment to residue 343 magenta) while Beta-49 HC (dark pink) and LC (blue) are shown as cartoons. The HC contacts two RBDs, forming a primary (circle) and secondary (ellipse) epitope. (D) Top view of the RBDs in all RBD down S (PDB 7NDA) and in the Beta-49 bound state. The 3-fold axis of S is shown. One RBD is superposed (reference), arrows show the movement in the other RBDs induced on binding Beta-49. (E) Close up of the secondary epitope with some RBD residues marked. (F) Close up of Beta-49/Beta S interaction. The RBD is shown as sticks and a surface (glycan at N343 as sticks only), and Fab as sticks colored by chain. (G) Similar to (F) but for Beta-50. (H and I) Comparison of the binding of Beta-27 with mAbs 150 and 222. (H) Residue 501 is highlighted on the RBD surface. (I) Side view of the right shoulder and neck of the RBD. Arrows show shifts due to repositioning the HC CDR3. (J) Comparison of the attachment of Beta-6 and -32 to the RBD with axes (left panel) showing difference in pose. (K) K484 is enclosed by the Beta-38 HC and LC CDR3s. See also Figure S4 and Tables S2 and S4.

Beta-49 and -50 potently neutralized all strains of SARS-CoV-2 tested (Figure S1D), bound tightly to the full S trimer but only very weakly to the RBD and did not block binding of ACE2 (Figures 1G and 1H). Both belong to the IgVH1-69 gene family, and we determined crystal structures of Fab/RBD complexes and cryo-EM Fab/S complexes for both (STAR Methods; Tables S2 and S4; Figures 4A and 4C). The relatively high resolution (2.8 Å) of the Beta-49 Fab/S structure (Figure 4C) suggested that the complex is rather rigid. Both mAbs attach in the same configuration, despite the presence of 19 amino acid differences in the variable domain of the heavy chain (VH) alone (Figure 6A; Table S3). Their epitope lies at the “waist” region of the RBD (Torso analogy Figures 3J and S4A) harboring the N- and C-terminal junctions of RBD with the rest of S1 and includes the N- and C-terminal residues of the RBD construct (Figure S4B). The N-linked glycan attached to residue 343 of the RBD also forms part of the epitope, the sugar becoming displaced from its usual position, twisting the sidechain of N343 into an unfavorable conformation (Figure 6B). Despite the sequence variation between the two mAbs the RBD interactions are very similar and involve conserved residues. A large portion of the HC interacting area is formed by the H3 loop (375 out of 632 Å^2^ for Beta-49). The Fab-bound S trimer shows all three RBDs in a down configuration (Figure 4C), with the HC making interactions with two RBDs (Figure 6C), causing the RBD to be translated/rotated toward the periphery of the trimer (Figure 6D). This “down and out” orientation has not, to the best of our knowledge, been seen before. The tight packing against the second RBD forms a secondary or quaternary epitope of some 210 Å^2^ (Figures 6C, 6E, and S4B). This secondary 1Fab:2RBD interaction, together with the fact that the primary epitope involves residues to the very ends of the soluble RBD construct likely explains the low affinity of Beta-49 and -50 to soluble monomeric RBD (Figure S4B). The residues comprising the primary (but not the secondary) epitope (Figures 6F and 6G) are conserved between SARS-CoV-1 and -2 (Figure S4B), and we were able to show (Figure S1B) that both mAbs bind SARS-CoV-1 and -2 S to a similar degree. Finally, Beta-49 and -50 potently neutralize a SARS-CoV-1 pseudovirus (Figure S1C).

### Limited use of cross-reactive members of the IgVH3-53/3-66 gene family

In contrast to the extensive use of public IgVH gene families to target Tyr-501, the well-known IgVH3-53/3-66 public antibody class (frequently elicited by the early pandemic virus) is represented by only Beta-27 in the set of most potent Beta neutralizers. This is explained by our previous observation that these mAbs are for the most part sensitive to mutation to Tyr at residue 501 of the RBD, while rare changes in the LC CDR1 can confer resilience. For example, in mAb-222 isolated from individuals infected with early pandemic strains, a proline is inserted at residue 30, which can pack against the Tyr-501 without the clashes engendered by most L1 sequences (Dejnirattisai et al., 2021b) allowing it to effectively neutralize all variants. The cryo-EM structure of Fab-222/Beta S shows that the majority of trimeric S particles were in the “2 RBD-up” configuration, with both upward RBDs engaging with 222-Fab in the mode expected from the earlier RBD/Fab complex structure (Figures 4C and 6H; Dejnirattisai et al., 2021b). This is in-line with the RBD “up” engagement pattern seen for other IgVH3-53/S complexes (Barnes et al., 2020; Dejnirattisai et al., 2021a).

Beta-27 uses an alternative mechanism to achieve potency against all variants. The Beta-27 H3 loop is lengthened to 11 residues from the usual 9, displacing the L1 to produce enough space to allow the large tyrosine side chain at 501 found in the Alpha, Beta, and Gamma variants to be stabilized by main chain peptide interactions at residues 29 and 30, analogous to mAb-222 (Figures 6H and 6I), conferring cross reactivity against all variants. As with canonical IgVH3-53 mAbs, Beta-27 makes extensive interactions with the RBD mainly in the neck region between the shoulders (Figure 4A, 718 Å^2^ with the HC and 262 Å^2^ with the LC) (Dejnirattisai et al., 2021a; Guo et al., 2021). H1-3 all contact the RBD surface in the area between, but not touching, residues 417 and 484. It appears from the relative rarity of IgVH3-53 mAbs in the anti-Beta responses, that the mAbs selected are private rather than public.

### Other gene families can attach strongly at the right shoulder around residue 501 while being cross-reactive

Beta-32 is highly potent against all variants reported here. We determined the high-resolution crystal structure of the Fab and the cryo-EM structure of the Fab in complex with Beta S (Figures 4A–4C and 6J). Fab binds to two RBDs in the up configuration. Although there is some ambiguity in the mode of association, it is clear that there are strong interactions centered on residue 501. The Beta-32-binding mode is radically different to that observed for the gene families IgVH4-39 and IgVH3-53 discussed earlier. Thus, the 501 interactions are via the HC instead of the LC as seen for the IgVH3-53 mAbs, and the angle of attack of Beta-32 compared with Beta-6 is slewed ∼90° toward the back of the RBD (Figure 6J). Thus, Beta-32 has found a cross-reactive mode of engagement with the right shoulder of the RBD.

### Exquisite specificity for Lys-484 can be achieved by a combination of a salt bridge and a hydrophobic cage

Beta-38 is classified by serology and BLI competition mapping as requiring Lys-484 (Figures 2C, 3I, and 3J; Table S1B). The Beta-38/Beta RBD complex structure confirms this (Figure 4A), with the antibody attacking the neck/left shoulder region from the front, achieving specificity by burying Lys-484 between the H3 and L3 loops (Figure 6K). The hydrophobic stem of the lysine side chain is contained within a hydrophobic cage composed of Phe-490 (RBD), Tyr-108 (HC), and Trp-92 (LC). At the end of the cage Asp-94 (LC) makes a salt bridge with the amino group of the lysine side chain of residue 484 (Figure 6K) and a hydrogen bond to Asn-32. There are two Fab-RBD complexes in the crystallographic asymmetric unit and the different crystal packing forces introduce a 13° difference in the angle of attack, indicating flexibility in the attachment of this highly focused antibody.

### Indirect effect of mutations at RBD residue 417

**Figure 7:**
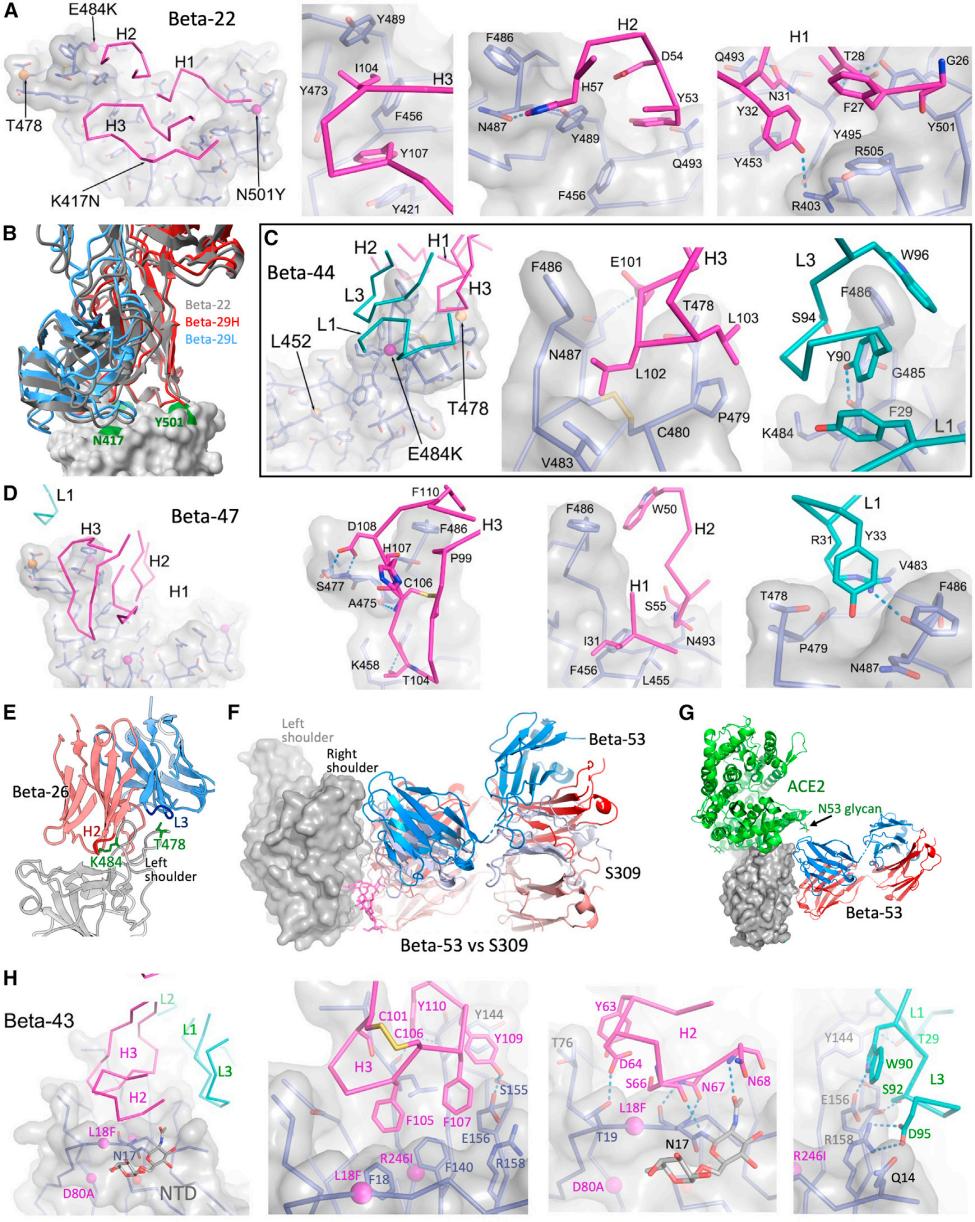
Details of interactions of Beta-22, -29, -47, -26, -53, and -43 (A) Interactions of Beta-22 (as Figure 6A). N417 specificity is achieved indirectly. (B) Identical binding modes of Beta-22 (gray) and Beta-29 (HC red, LC blue) IgVH3-30 mAbs. (C) Beta-44/Beta RDB interactions. (D) Beta-47/Beta RDB interactions. (E) Beta-26 binds the left shoulder contacting K484 and T478 of the RBD. (F) Beta-53 (HC red, LC blue) binds the same epitope as S309 (HC salmon, LC pale blue; PDB: 7BEP). (G) Binding of Beta-53 relative to ACE2 receptor. (H) Beta-43 binding to the NTD (gray surface). See also Figure S5.

Beta-22 uses IgVH3-30 and is classified serologically as targeting residue 417 (Figure 2D; Table S1B). The 417 focus is reinforced by the BLI mapping, which places the antibody almost exactly atop this residue (Figure 3J), and the crystal structure of the complex confirms this (Figures 4A and 7A). However, the binding is quite extensive, almost entirely restricted to the HC, with H1-3 contributing a total of 597 Å^2^ interface area (in contrast the LC contributes only 110 Å^2^). H1-3 are deployed so that H1 is close to residue Tyr-501, H3 wraps across Asn-417 and H2 reaches up toward residue Lys-484 (Figure 7A).

The serological data shows good correlation with this (Figure 2D), H2 fails to reach Lys-484 hence this mutation has little impact on binding, whereas the N501Y mutation has a positive impact on binding but K417N/T is also required for effective neutralization. Although Asn-417 does not make direct contacts with H3 the extra size of the lysine and the concentration of positive electrostatic charge presumably combine to have a significant effect on antibody affinity. Beta-22 is glycosylated at residue Asn-35 within L1 and the sugar, as observed before, lies in the vicinity of the left shoulder (Dejnirattisai et al., 2021a). There are two IgVH3-30 mAbs, isolated from different donors (Table S1B), in the set of potent Beta RBD binders, the second being Beta-29. Serology and BLI mapping suggest that Beta-29 is similar to Beta-22, and the crystal structure of the Beta-29/Beta RBD complex confirmed essentially identical binding modes (Figure 7B). Interestingly, these two mAbs also share IgVL4-1. Although we have no structural information for the third potent mAb sharing these serological properties, Beta-20, the BLI competition mapping places this mAb in an identical position and the HC gene family IgVH3-33 is closely related to IgVH3-30 (Table S3), so it is possible that the mode of engagement may be similar (although the NTD binder Beta-43 is also IgVH3-30). In summary, specificity for Asn-417 seems to be achieved indirectly, with the residue nestled at the heart of the antibody-binding site between L2 and H3. Although there are no specific high-affinity interactions, placing the residue central to the paratope renders it sensitive to the change in electrostatic charge at 417 characteristic of the Alpha, Beta, and Gamma variants, but agnostic to the residue being an asparagine or threonine. It may be that Beta-20, which is sensitive to Thr-417, makes direct contact with Asn-417.

### Targeting the left shoulder can introduce sensitivity to Delta through residue 478

Beta-44 does not neutralize the Delta variant. This mAb is perched on the left shoulder making relatively small contacts via both HC and LC (408 and 123 Å^2^, respectively), with the HC positioned above residue 478 (Figure 4A). L1 and L3 are close to residue 484 of the RBD, making a hydrogen bond from the hydroxyl group of Tyr-90 of the L3 loop to the carbonyl oxygen of Lys-484, while H1, H2, and H3 surround residue Thr-478 (Figure 7C). Since there are no contacts close to Leu-452 of the RBD, sensitivity to Delta arises through contacts with residue 478, perhaps due to loss of hydrophobic interactions between the side chain CG1 and H3 residue Tyr-90 when residue 478 is mutated to Lys (Figure 7C). Cryo-EM analysis of the Beta-44-Fab/Beta S complex shows two RBDs in the up configuration with Fab attached (Figure 4C).

### Cross reactivity can be obtained by binding the left shoulder-neck region but avoiding variant mutations

Beta-47 is cross-reactive against all variants and interacts strongly with the back of the left shoulder-neck interface (contact areas are 582 and 234 Å^2^ for the HC and LC, respectively) (Figures 4A and 7D). H3 makes the largest contact, in part to the back of the loop bearing 484, whereas LC contacts are to the far edge of the left shoulder, in the vicinity of, but not contacting, residue 478 (Figure 7D). Beta-47 is also glycosylated, at residue Asn-102 of H3, as for Beta-22 the sugar lies in the vicinity of the left shoulder but makes little specific contact with the RBD. Beta-47 is very similar to mAb 253 previously identified (Dejnirattisai et al., 2021a), sharing the same variable genes, glycosylation, and a disulphide in the H3.

### Targeting the left shoulder can produce sensitivity to changes at residues 484 and 478

MAb Beta-26 requires the Beta E484K mutation for potent neutralization but is also exquisitely sensitive to the L452R/T478K mutations found in Delta. To investigate this, we determined the cryo-EM Beta S/Beta-26 Fab structure. Unusually, S was found in a 3 RBD-up configuration with Fab attached to all three RBDs (Figure 4C). Since the experimental structure was insufficiently resolved to build the Fab structure directly, we constructed a model with Alphafold-2 (Jumper et al., 2021), and adjusted it to fit the density (STAR Methods). The major contacts are through the H1–3, with H2 making a close interaction with residue 484 (Figure 7E). In addition, there are strong interactions between L3 and 478 (Figure 7E). These interactions explain the observed serological results (Figure 2C; Table S1A).

### A potently neutralizing cross-reactive epitope that does not block ACE2 binding

BLI competition analysis placed the potent cross-reacting antibody Beta-53 (Table S1A) on the upper right flank of the RBD (Figure 3J), and ELISA data showed that binding was independent of ACE2 binding (Figure 1G). The Beta-53/Beta S complex structure shows attachment to all three RBDs (2 up and one down) (Figure 4C), and higher resolution analysis of the Beta-53/Beta RBD complex confirms that the antibody attaches at an epitope overlapping that of antibody S309 identified earlier (Figure 7F; Pinto et al., 2020). There is also some overlap with the Beta-49 and -50 “waist”-binding mAbs described above (Figure S4A). Like S309, Beta-53 makes a substantial interaction with the N-linked glycan at residue N343 (Figures 6B, 6F, 6G, and 7F). Compared with S309, Beta-53 sits some 10 Å further up the RBD, toward the ACE2-binding site so that Beta-53 contacts the glycans via H1 and H3 instead of H3 and L2 in S309. It is even further from the Beta-49/Beta-50 “waist” epitope. The Fab approaches the ACE2 site and is likely to brush against the N53 ACE2 glycan (Figure 7G); however, ELISA competition data confirm that there is no significant competition with ACE2 (Figure 1G). Both the HC and LC make substantial contacts (466 and 270 Å^2^, respectively).

The Beta-53/Beta S complex structure determined after incubation at 20°C for 30 min may cast light on the mechanism of neutralization. Despite the incubation temperature being below physiological, a substantial fraction of S was no longer trimeric, suggesting S destruction as a potential neutralization mechanism, as proposed for other non-ACE2-competing mAbs (STAR Methods) (Huo et al., 2020; Yuan et al., 2020b; Zhou et al., 2020).

### A Beta-specific NTD-binding antibody

Beta-43 is the only potent non-RBD mAb described here and is highly specific to Beta S (Figure S1A). X-ray and EM analyses confirm direct interaction with the NTD, at the so-called “supersite” (Figures 4A, 4C, 7H, and S5; (Cerutti et al., 2021; McCallum et al., 2021). Of the point mutations in the NTD (L18F, D80A, D215G, and R246I) only L18F is part of the epitope. Although the epitope is slightly removed from the site of the Beta characteristic three-residue (242–244) NTD deletion, comparison of the Beta and early pandemic NTD structures reveals a knock-on change that significantly shifts part of the Beta-43 epitope, conferring Beta specificity (Figure S5). In addition to this variant-specific conformational change, there are numerous other differences between the Beta-NTD and other reported NTD structures (Figure S5), highlighting the intrinsic flexibility of this highly mutable domain.

## Discussion

Following Beta infection, there is a marked shift in the profile of the antibody response compared with infection with early pandemic strains (Figures 3H and 3I), with many potent mAbs picking out the three RBD amino acid changes found in Beta; K417N (3/27 mAbs), E484K (6/27 mAbs), and especially N501Y (11/27 mAbs) (Greaney et al., 2021). This specificity underpins the antigenic difference between Beta, the other VoCs, and early pandemic strains/vaccines. Of the 27 mAbs, titers were reduced compared with Beta by 1 log, 2 logs, or knocked out (KO), respectively for Victoria (18, 15, 10), Alpha (10, 6, 5), Gamma (2, 1, 1), and Delta (18, 16, 14). These profound reductions in the neutralization potential of Beta-specific mAbs underscore the antigenic distance between Beta and early pandemic strains (10/27 mAbs KO), which is even more extreme with Delta (14/27 mAbs KO). Delta differs from Beta by 5 amino acids in the RBD (K417, L452, T478, E484, N501), while Beta and Gamma are antigenically close (1/27 mAbs KO), finally Alpha, which contains the single N501Y mutation, occupies an intermediate position (5/27 mAbs KO). These data are consistent with neutralization data using Beta and Gamma serum, which have much reduced neutralization capacity for Delta with many completely failing to neutralize Delta (Liu et al., 2021).

Part of the reason for the shift of the mAb response to Beta compared with early pandemic strains is the response to Tyr501 (11/27 mAbs). A significant portion of this is driven by the activation of a public antibody response to N501Y through IgVH4-39 (6/11 N501Y specific mAbs), and the highly similar IgVH4-30 (1/11). As expected for a public response, these mAbs engage the same epitope in a closely related way, with different lengths of H3 being accommodated by a pivoting of the antibody around residue 501, shifting the positions of the H1 and H2 loops, which appear to “rachet” between two stable sets of interactions (Figures 5B–5D; Table S1B).

Despite many mAbs derived from Beta cases showing specificity to a subset of VoCs, 6/27 mAbs showed potent neutralization of all VoCs tested (<100 ng/mL). Two of these belong to public responses that have been repeatedly isolated (mAb 27 IgVH3-53 and mAb 45 IgVH1-58). The continued, but much reduced, usage of the IgVH3-53 gene family is due to a shift in L1 abrogating a blocking interaction with Tyr-501 bearing RBDs (Figure 6H) and joins the use of a rare mutation in L1 documented previously (Dejnirattisai et al., 2021b) as a resilience mechanism against this mutation.

Another pair of potent cross-reactive mAbs (Beta-49 and -50) belong to the IgVH1-69 gene family and bind the “waist” of the RBD, to a previously undetected epitope conserved in SARS-CoV-1 (Figure S4). It is possible that the mechanism of neutralization may be to lock the RBDs in an unusual “down and out” conformation.

The potently neutralizing NTD-binding antibody, Beta-43, binds the NTD in the so-called supersite region (Cerutti et al., 2021). This antibody is specific for the Beta variant, and the Fab/NTD complex structure shows how this specificity is achieved, the three-residue deletion in Beta (Δ242–244) causes a conformation change that impacts on the supersite and will likely cause more generalized escape from supersite binders than the individual amino acid changes also found in Beta NTD (D80A, D215G, and R246F).

One potent neutralizer, Beta-53, remains a puzzle. It does not compete with ACE2, binds an epitope overlapping that of S309 (Pinto et al., 2020), and similar to S309 it interacts with the N-linked glycan at residue N343 of the RBD. The mechanism of neutralization is unclear; however, there is a suggestion from the cryo-EM of the complex of Beta-53 Fab with the trimeric S protein, that at room temperature the Fab may destabilize the prefusion state of S.

In summary, we have presented an in-depth structure-function analysis of potent mAbs from Beta-infected volunteers. Looking at potent neutralizing mAbs, the anti-Beta response is substantially repositioned toward the three mutated residues found in the Beta RBD. Differential targeting of these residues creates the large antigenic distance between Beta and early pandemic strains, which are used in current vaccines. The majority of Beta-specific mAbs fail to neutralize Delta, which is consistent with the large falls in the ability of Beta (and Gamma) infected sera to neutralize Delta (Liu et al., 2021) and reflects the fact that there are 5 amino acid differences between the RBDs of Beta and Delta. Several companies are now developing Beta booster vaccines, and it will be interesting to determine how effective they are at eliciting responses to the key mutated residues in Beta in individuals vaccinated with Wuhan related strains. Finally, it seems likely that further VoCs will occur in the future by *de novo* mutation or through recombination, whether we see the emergence of widespread escape from vaccines mandating a change in strategy toward polyvalent vaccination, as seen with influenza, or a search for broadly neutralizing monovalent vaccines remains to be determined.

### Limitations of the study

The neutralization assays described are performed *in vitro* and therefore do not capture the contribution of complement or ADCC, which may augment responses *in vivo*. They also do not take account of T cell responses, which are not disrupted to the same degree as the antibody response by the relatively small number of mutations occurring in S compared with its overall length (1,273 amino acids), T cells may play a role in protection from severe disease (Fischer et al., 2021).

It will be interesting to survey mAb responses following Delta infection and see whether the large reductions or failure of Beta and Gamma serum to neutralize Delta is replicated when Delta serum is used to neutralize Beta and Gamma.

## STAR★Methods

### Key resources table

**Table:** 

REAGENT or RESOURCE	SOURCE	IDENTIFIER
**Antibodies**		

Fab	This paper	N/A
IgG	This paper	N/A
Human anti-NP (mAb 206)	Dejnirattisai et al., 2021a	N/A
Human anti-NTD (mAb407)	Dejnirattisai et al., 2021a	N/A
Human anti-RBD (mAb222)	Dejnirattisai et al., 2021a	N/A
S309	LifeArc	N/A
StrepMAB-Classic	IBA Lifesciences	Cat# 2-1507-001
StrepMAB Classic DY549	IBA Lifesciences	Cat#2-1566-050
Anti-Human CD3-FITC	BD Biosciences	Cat# 555332
Anti-Human CD14-FITC	BD Biosciences	Cat# 555397
Anti-Human CD56-FITC	BD Biosciences	Cat# 562794
Anti-Human CD16-FITC	BD Biosciences	Cat# 555406
Anti-Human IgM-FITC	BD Biosciences	Cat# 555782
Anti-Human CD19-BUV395	BD Biosciences	Cat# 563549
Anti-Human IgG-BV786	BD Biosciences	Cat# 564230
Anti-IgM-APC	BD Biosciences	Cat# 551062
Anti-Human IgA-FITC	Dako	Cat# F0188
Anti-Human IgD-FITC	Dako	Cat# F0189
Anti-Human IgG (Fab-specific)-ALP	Sigma-Aldrich	Cat# A8542
Anti-Human IgG (Fc-specific)-ALP	Sigma-Aldrich	Cat# A9544
Anti-Human IgG (Fc specific)-Peroxidase	Sigma-Aldrich	Cat# A0170
Anti-human IgG Fc specific-FITC	Sigma-Aldrich	Cat# F9512

**Bacterial and virus strains**		

SARS-CoV-2 (Australia/VIC01/2020)	Caly et al., 2020	N/A
SARS-CoV-2 strain Alpha	Public Health England	N/A
SARS-CoV-2 strain Beta	Public Health England	N/A
SARS-CoV-2 strain Gamma	Dejnirattisai et al., 2021a	N/A
SARS-CoV-2 strain Alpha+E484K	This paper	N/A
SARS-CoV-2 strain Delta	Wendy Barclay and Thushan De Silva	N/A
SARS-CoV-2/B.1.525	Wendy Barclay and Thushan De Silva	N/A
DH5α bacteria	Invitrogen	Cat# 18263012

**Biological samples**		

PBMCs from SARS-CoV-2 patients	John Radcliffe Hospital in Oxford UK	N/A
Chemicals, Peptides, and Recombinant Proteins		
Phosphate buffered saline tablets	Sigma-Aldrich	Cat. No. P4417
Sensor Chip Protein A	Cytiva	Cat#29127555
Dulbecco’s Modified Eagle Medium, high glucose	Sigma-Aldrich	Cat# D5796
Dulbecco’s Modified Eagle Medium, low glucose	Sigma-Aldrich	Cat# D6046
UltraDOMA PF Protein-free Medium	Lonza	Cat#12-727F
FreeStyle™ 293 Expression Medium	Gibco	Cat# 12338018
L-Glutamine–Penicillin–Streptomycin solution	Sigma-Aldrich	Cat# G1146
GlutaMAX™ Supplement	Gibco	Cat#35050061
Fetal Bovine Serum	Gibco	Cat# 12676029
TrypLE™ Express Enzyme	Gibco	Cat#12604013
Trypsin-EDTA	Gibco	Cat#2259288
Opti-MEM	Gibco	Cat# 11058021
Polyethylenimine, branched	Sigma-Aldrich	Cat# 408727
His-tagged SARS-CoV-2 RBD K417N, E484K, N501Y	Zhou et al., 2021	N/A
SARS-CoV-2 Beta variant Spike	This paper	N/A
SARS-CoV Spike	Dejnirattisai et al., 2021a	N/A
His-tagged human ACE2	Dejnirattisai et al., 2021a	N/A
His-tagged SARS-CoV-2 NTD	This paper	N/A
His-tagged SARS-CoV-2 alpha variant NTD	This paper	N/A
His-tagged SARS-CoV-2 beta variant NTD	This paper	N/A
His-tagged SARS-CoV-2 gamma variant NTD	This paper	N/A
His-tagged SARS-CoV-2 delta variant NTD	This paper	N/A
Streptavidin-APC	Biolegend	Cat# 405207
RNase inhibitor	Promega	Cat# N2611
Carboxymethyl cellulose	Sigma-Aldrich	Cat# C4888
Strep-Tactin®XT	IBA Lifesciences	Cat# 2-1206-025
Triton X-100	Sigma-Aldrich	Cat#8787
Protein G Plus/Protein A Agarose	Millipore	Cat#IP10

**Critical commercial assays**		

Bright-Glo Luciferase Assay System	Promega	Cat# E2620
HIV Type 1 p24 Antigen ELISA 2.0	ZeptoMetrix	Cat# 0801002

**Deposited data**		

Crystal structure of SARS-CoV-2 Beta-RBD/Beta-6 and COVOX-45 Fab complex	This paper	PDB: 7PRY
Crystal structure of SARS-CoV-2 Beta-RBD/Beta-22 Fab complex	This paper	PDB: 7PRZ
Crystal structure of SARS-CoV-2 Beta-RBD/Beta-24 Fab complex	This paper	PDB: 7PS0
Crystal structure of SARS-CoV-2 Beta-RBD/Beta-27 Fab complex	This paper	PDB: 7PS1
Crystal structure of SARS-CoV-2 Beta-RBD/Beta-29 and Beta-53 Fab complex	This paper	PDB: 7PS2
Crystal structure of SARS-CoV-2 Beta-RBD/Beta-38 Fab complex	This paper	PDB: 7PS4
Crystal structure of Beta-32 Fab	This paper	PDB: 7PS3
Crystal structure of SARS-CoV-2 Beta-RBD/Beta-40 Fab complex	This paper	PDB: 7PS7
Crystal structure of SARS-CoV-2 Beta-RBD/Beta-47 Fab complex	This paper	PDB: 7PS5
Crystal structure of SARS-CoV-2 Beta-RBD/Beta-44 and Beta-54 Fab complex	This paper	PDB: 7PS6
Crystal structure of SARS-CoV-2 Beta-RBD/Beta-49 and FI3A Fab complex	This paper	PDB: 7Q0G
Crystal structure of SARS-CoV-2 Beta-RBD/Beta-50 and Beta-54 Fab complex	This paper	PDB: 7Q0H
Crystal structure of SARS-CoV-2 Beta-RBD/Beta-43 Fab complex	This paper	PDB: 7Q0I
CryoEM structure of COVOX-222 Fab in complex with SARS-CoV-2 Beta Spike glycoprotein trimer	This paper	EMD-13869, PDB: 7Q9G
CryoEM structure of Beta-49 Fab in complex with SARS-CoV-2 Beta Spike glycoprotein trimer	This paper	EMD-13857, PDB: 7Q9E
CryoEM structure of Beta-50 Fab in complex with SARS-CoV-2 Beta Spike glycoprotein trimer	This paper	EMD-13868, PDB: 7Q9F
CryoEM structure of Beta-43 Fab in complex with SARS-CoV-2 Beta Spike glycoprotein trimer	This paper	EMD-13870, PDB: 7Q9I
CryoEM structure of Beta-32 Fab in complex with SARS-CoV-2 Beta Spike glycoprotein trimer	This paper	EMD-13872, PDB: 7Q9K
CryoEM structure of Beta-26 Fab in complex with SARS-CoV-2 Beta Spike glycoprotein trimer	This paper	EMD-13871, PDB: 7Q9J
CryoEM structure of Beta-53 Fab in complex with SARS-CoV-2 Beta Spike glycoprotein trimer	This paper	EMD-13873, PDB: 7Q9M
CryoEM structure of Beta-06 Fab in complex with SARS-CoV-2 Beta Spike glycoprotein trimer	This paper	EMD-13875, PDB: 7Q9P
CryoEM map of Beta-44 Fab in complex with SARS-CoV-2 Beta Spike glycoprotein trimer	This paper	EMD-13874

**Experimental models: Cell lines and animals**		

HEK293S GnTI- cells	ATCC	Cat# CRL-3022
HEK293 cells	ATCC	Cat# CRL-3216
Expi293F™ Cells	Gibco	Cat# A14527
HEK293T/17 cells	ATCC	Cat#CRL-11268
HEK293T/17 cells-hACE2	Liu et al., 2021	N/A
Hamster: ExpiCHO cells	Thermo Fisher	Cat# A29133
Vero cells	ATCC	Cat# CCL 81
VeroE6/TMPRSS2	NIBSC	Cat#100978
Heterozygous K18-hACE C57BL/6J mice, 7-8 week-old	Jackson Laboratory	2B6.Cg-Tg(K18-ACE2)2Prlmn/J)

**Recombinant DNA**		

Vector: pHLsec	Aricescu et al., 2006	N/A
Vector: pOPING-ET	Nettleship et al., 2008	N/A
Vector: p8.91	di Genova et al., 2020	Nigel Temperton
Vector: pCSFLW	di Genova et al., 2020	Nigel Temperton
Vector: pcDNA-SARS-CoV spike	Nigel Temperton	N/A
Vector: pcDNA-SARS-CoV-2 spike of Beta strain	This paper	N/A
human ACE2 cDNA	Sourcebiosciences	ID: 5297380
Vector: human IgG1 heavy chain	German Cancer Research Center, Heidelberg, Germany (H. Wardemann	N/A
Vector: human lambda light chain	German Cancer Research Center, Heidelberg, Germany (H. Wardemann	N/A
Vector: human kappa light chain	German Cancer Research Center, Heidelberg, Germany (H. Wardemann	N/A
Vector: Human Fab	University of Oxford, NDM (G. Screaton)	N/A
TM149 BirA pDisplay	University of Oxford, NDM (C. Siebold)	N/A

**Software and algorithms**		

Xia2-dials	Winter et al., 2018	https://xia2.github.io/parameters.html
PHENIX	Liebschner et al., 2019	https://www.phenix-online.org/
COOT	Emsley and Cowtan, 2004	https://www.mrc-lmb.cam.ac.uk/personal/pemsley/coot/
Data Acquisition Software 11.1.0.11	Fortebio	https://www.fortebio.com/products/octet-systems-software
Data Analysis Software HT 11.1.0.25	Fortebio	https://www.fortebio.com/products/octet-systems-software
CryoSPARC v2.15.1-live	Structura Biotechnology Inc.	https://cryosparc.com/
SerialEM (version 3.8.0 beta)	https://bio3d.colorado.edu/SerialEM/; Mastronarde, 2005	N/A
EPU	Thermo Fisher	https://www.thermofisher.com/uk/en/home/electron-microscopy/products/software-em-3d-vis/epu-software.html
Prism 8.0	GraphPad	https://www.graphpad.com/scientific-software/prism/
Biacore T200 Evaluation Software 3.1	Cytiva	www.cytivalifesciences.com
IBM SPSS Software 26	IBM	https://www.ibm.com
Flowjo 10.7.1	BD	https://www.flowjo.com
SnapGene software 5.3.2	Insightful Science	www.snapgene.com

**Other**		

X-ray data were collected at beamline I03, Diamond Light Source, under proposal lb27009 for COVID-19 rapid access	This paper	https://www.diamond.ac.uk/covid-19/for-scientists/rapid-access.html
Cryo-EM data were collected at eBIC, Diamond, under Proposal **BI26983-2** for COVID-19 rapid access	This paper	https://www.diamond.ac.uk/covid-19/for-scientists/rapid-access.html
Cryo-EM data were collected at OPIC, Division of Structural Biology, University of Oxford	This paper	https://www.opic.ox.ac.uk/
HiLoad® 16/600 Superdex® 200 pg	Cytiva	Cat. No. 28-9893-35
Amine Reactive Second-Generation (AR2G) Biosensors	Fortebio	Cat# 18-5092
Octet RED96e	Fortebio	https://www.fortebio.com/products/label-free-bli-detection/8-channel-octet-systems
QuixStand	GE Healthcare	Cat# 56-4107-78

### Resource availability

#### Lead contact

Resources, reagents and further information requirement should be forwarded to and will be responded by the Lead Contact, David I Stuart (dave@strubi.ox.ac.uk).

#### Materials availability

Reagents generated in this study are available from the Lead Contact with a completed Materials Transfer Agreement.

### Experimental model and subject details

#### Viral stocks

SARS-CoV-2/human/AUS/VIC01/2020 (Caly et al., 2020) and SARS-CoV-2/Beta, provided by Public Health England, were both grown in Vero (ATCC CCL-81) cells. Cells were infected with the SARS-CoV-2 virus using an MOI of 0.0001. Virus containing supernatant was harvested at 80% CPE and spun at 2000 rpm at 4 °C before storage at -80 °C. Viral titres were determined by a focus-forming assay on Vero cells. Both Victoria passage 5 and Beta passage 4 stocks were sequenced to verify that they contained the expected S sequence and no changes to the furin cleavage sites. The Beta virus used in these studies contained the following mutations: D80A, D215G, L242-244 deleted, K417N, E484K, N501Y, D614G, A701V.

#### Bacterial strains and cell culture

Vero (ATCC CCL-81) cells were cultured at 37 °C in Dulbecco’s Modified Eagle medium (DMEM) high glucose (Sigma-Aldrich) supplemented with 10% fetal bovine serum (FBS), 2 mM GlutaMAX (Gibco, 35050061) and 100 U/ml of penicillin–streptomycin. Human mAbs were expressed in HEK293T cells cultured in UltraDOMA PF Protein-free Medium (Cat# 12-727F, LONZA) at 37 °C with 5% CO_2_. *E.coli DH5α* bacteria were used for transformation of plasmid pNEO-RBD K417N, E484K, N501Y. A single colony was picked and cultured in LB broth with 50 μg mL^-1^ Kanamycin at 37 °C at 200 rpm in a shaker overnight. HEK293T (ATCC CRL-11268) cells were cultured in DMEM high glucose (Sigma-Aldrich) supplemented with 10% FBS, 1% 100X Mem Neaa (Gibco) and 1% 100X L-Glutamine (Gibco) at 37 °C with 5% CO_2_. To express RBD, RBD K417N, E484K, N501Y and ACE2, HEK293T cells were cultured in DMEM high glucose (Sigma) supplemented with 2% FBS, 1% 100X Mem Neaa and 1% 100X L-Glutamine at 37 °C for transfection.

#### Sera from Beta Infected Cases

Beta samples from UK infected cases were collected under the “Innate and adaptive immunity against SARS-CoV-2 in healthcare worker family and household members” protocol affiliated to the Gastro-intestinal illness in Oxford: COVID sub study discussed above and approved by the University of Oxford Central University Research Ethics Committee. All individuals had sequence confirmed Beta infection or PCR-confirmed symptomatic disease occurring whilst in isolation and in direct contact with Beta sequence-confirmed cases. Additional Beta infected serum (sequence confirmed) was obtained from South Africa. The potent antibodies analysed here derived from 4 male patients with ages in the range 40-64. At the time of swab collection patients signed an informed consent to consent for the collection of data and serial blood samples. The study was approved by the Human Research Ethics Committee of the University of the Witwatersrand (reference number 200313) and conducted in accordance with Good Clinical Practice guidelines.

#### Mouse experiments

Animal studies were carried out in accordance with the recommendations in the Guide for the Care and Use of Laboratory Animals of the National Institutes of Health. The protocols were approved by the Institutional Animal Care and Use Committee at the Washington University School of Medicine (assurance number A3381–01). Virus inoculations were performed under anaesthesia that was induced and maintained with ketamine hydrochloride and xylazine, and all efforts were made to minimize animal suffering.

Heterozygous K18-hACE C57BL/6J mice (strain: 2B6.Cg-Tg(K18-ACE2)2Prlmn/J). Animals were housed in groups and fed standard chow diets. Mice of different ages and both sexes were administered 10^3^ FFU of SARS-CoV-2 via intranasal administration.

For the mouse experiments Vero-hACE2-TMPRSS2 (a gift of A. Creanga and B. Graham, NIH) and Vero-TMPRSS2 cells were cultured at 37°C in Dulbecco’s Modified Eagle medium (DMEM) supplemented with 10% fetal bovine serum (FBS), 10 mM HEPES pH 7.3, 1 mM sodium pyruvate, 1× non-essential amino acids, and 100 U/ml of penicillin–streptomycin. Additionally, Vero-TMPRSS2 and Vero-hACE2-TMPRSS2 cells were cultured in the presence of 5 μg/mL of blasticidin or puromycin, respectively. The Beta SARS-CoV-2 strain was obtained from a nasopharyngeal isolate (a gift of M. Suthar, Emory). Infectious stocks were propagated by inoculating Vero-TMPRSS2 cells. Supernatant was collected, aliquoted, and stored at -80^o^C. All work with infectious SARS-CoV-2 was performed in Institutional Biosafety Committee-approved BSL3 and A-BSL3 facilities at Washington University School of Medicine using positive pressure air respirators and protective equipment. Viral sequence was confirmed by deep-sequencing after RNA extraction to confirm the presence of the anticipated substitutions.

### Method details

#### Beta S Protein

To construct the expression plasmids for the S protein of Beta, a construction of trimeric S of the Wuhan strain was used as the template (Dejnirattisai et al., 2021a) and nine pairs of primers of S (L18F forward primer 5’-GAGCAGCCAGTGCGTGAATTTCACCACCAGAACCCAGCTG-3’, L18F reverse primer 5’-CAGCTGGGTTCTGGTGGTGAAATTCACGCACTGGCTGCTC -3’; D80A forward primer 5’-GCACCAAGAGATTCGCCAATCCTGTGCTGCC-3’ and D80A reverse primer 5’-GGCAGCACAGGATTGGCGAATCTCTTGGTGC-3’; D215G forward primer 5’-ATTAATCTGGTGAGAGGCCTGCCTCAGGGCTTC-3’, D215G reverse primer 5’-GAAGCCCTGAGGCAGGCCTCTCACCAGATTAAT-3’; 242-244 deletion and R246I forward primer 5’-CCAGATTCCAGACCCTGCACATATCATATCTTACACCAG-3’, 242-244 deletion and R246I reverse primer 5’-CTGGTGTAAGATATGATATGTGCAGGGTCTGGAATCTGG-3′; K417N forward primer 5’-CAGGGCAGACCGGCAATATCGCCGACTACAATTAC-3’, K417N reverse primer 5’-GTAATTGTAGTCGGCGATATTGCCGGTCTGCCCTG -3’; E484K forward primer 5’-CACCGTGTAATGGCGTGAAGGGCTTCAATTGCTAC-3’, E484K reverse primer 5’-GTAGCAATTGAAGCCCTTCACGCCATTACACGGTG-3’; N501Y forward primer 5’-GCTTCCAGCCTACCTATGGCGTGGGCTAC-3’, N501Y reverse primer 5’-GTAGCCCACGCCATAGGTAGGCTGGAAGC-3’; D614G forward primer 5’-GCCGTGCTGTACCAGGGCGTGAATTGCACCGAG-3’, D614G reverse primer 5’-CTCGGTGCAATTCACGCCCTGGTACAGCACGGC-3’; A701V forward primer 5’-CACCATGAGCCTGGGCGTCGAGAATAGCGTGGCC-3’, A701V reverse primer 5’-GGCCACGCTATTCTCGACGCCCAGGCTCATGGTG-3’) and two primers of pHLsec vector (pHLsec forward primer 5’-CCTCAATTTGAGAAATAATGACTCGAGACTAGTATCGCG-3’, pHLsec reverse primer 5’-CGCGATACTAGTCTCGAGTCATTATTTCTCAAATTGAGG-3’) were used to do PCR. Amplified PCR fragments were joint together by Gibson reaction (Gibson, 2011). The new construct was fully sequenced.

#### Cloning of ACE2 and RBD K417N, E484K, N501Y

ACE2 and RBD K417N, E484K, N501Y were constructed as previously described (Zhou et al., 2021). Briefly, ACE2 was constructed by amplifying amino acids 19-615 of the human ACE2 from an image clone (Sourcebiosciences, clone ID: 5297380) using the forward primer 5’- GCGTAGCTGAAACCGGCTCCACCATTGAGGAACAGGCC-3’ and the reverse primer 5’-GTGATGGTGATGTTTGTCTGCATATGGACTCCAGTC-3’ and inserted into the vector pOPINTTGneo incorporating a C-terminal 6×His tag.

To construct RBD K417N, E484K, N501Y, the RBD N501Y construct was used as the template and K417N primers (Forward 5’-CAGGGCAGACCGGCAATATCGCCGACTACAATTAC-3’, Reverse 5’-GTAATTGTAGTCGGCGATATTGCCGGTCTGCCCTG-3’), E484K primers (Forward 5’- CACCGTGTAATGGCGTGAAGGGCTTCAATTGCTAC-3’, Reverse 5’- GTAGCAATTGAAGCCCTTCACGCCATTACACGGTG-3’) and primers of pNEO vector (Forward 5’- CAGCTCCTGGGCAACGTGCT-3’ and Reverse 5’-CGTAAAAGGAGCAACATAG-3’) were used to amplify DNA fragments. Three PCR fragments were used as templates and amplified again using pNEO vector primers. The final PCR fragment was digested by restriction enzymes AgeI and KpnI and ligated into digested pNEO vector. This construct was confirmed by sequencing.

#### Cloning of NTD of VoCs

To construct NTD of all VoCs, the gene encoding amino acids 13-305 of the NTD of SARS-CoV-2 S was amplified from soluble S plasmid of each variant by using the forward primer 5′- GGTTGCGTAGCTGAAACCGGTACCATGTTCGTGTTCCTGGTGCTGC -3′ and the reverse primer 5′-TTAGTGATGGTGATGGTGATGTTTTTCATGCCATTCAATCTTTTGTGCCTCAAAAATATCATTCAAGCTCTTCAGGGTGCACTTGGTCTC -3′ that has a C-terminal BirA-6xHis tag sequence. First PCR products were used as the template to amplify NTD gene, BirA-6xHis tag and overlapping sequence for Gibson assembly by using forward primer 5’- GATGGGTTGCGTAGCTGAAACCGGTAGTCAGTGTGTTAATCTTACAACCAGAACTCAATTAC-3’ for Wuhan and alpha, forward primer 5’- GATGGGTTGCGTAGCTGAAACCGGTAGCCAGTGCGTGAATTTCACC-3’ for gamma, forward primer 5’- GATGGGTTGCGTAGCTGAAACCGGTAGTCAGTGTGTTAATCTTAGGACCAGAACCCAG-3’ fo delta and reverse primer 5’- GATGGGTTGCGTAGCTGAAACCGGTAGCCAGTGCGTGAATTTCACC-3’ for all variants. The second PCR products were cloned into the pHLsec expression vector using the AgeI and XhoI restriction sites. All vectors were sequenced to confirm clones were correct by Sanger sequencing.

#### Protein Production

Protein expression and purification were conducted as described previously (Dejnirattisai et al., 2021a; Zhou et al., 2020). Briefly, plasmids encoding proteins were transiently expressed in HEK293T (ATCC CRL-11268) cells. The conditioned medium was dialysed and purified with a 5 mL HisTrap nickel column (GE Healthcare) and further polished using a Superdex 75 HiLoad 16/60 gel filtration column (GE Healthcare).

#### Isolation of Beta S-Specific Single B Cells by FACS

Beta S-specific single B cell sorting was performed as previously described (Dejnirattisai et al., 2021a). Briefly, PBMC were stained with LIVE/DEAD Fixable Aqua dye (Invitrogen) followed by recombinant trimeric S-twin-Strep of Beta. Cells were then incubated with CD3-FITC, CD14-FITC, CD16-FITC, CD56-FITC, IgM-FITC, IgA-FITC, IgD-FITC, IgG-BV786 and CD19-BUV395, along with Strep-MAB-DY549 to stain the twin strep tag of the S protein. IgG+ memory B cells were gated as CD19+, IgG+, CD3-, CD14-, CD56-, CD16-, IgM-, IgA- and IgD-, and S+ was further selected and single cells were sorted into 96-well PCR plates with 10 μl of catching buffer (Tris, Nuclease free-H2O and RNase inhibitor). Plates were briefly centrifuged at 2000ⅹg for 1 min and left on dry ice before being stored at -80 °C.

#### Cloning and Expression of Beta S-Specific Human mAbs

Beta S-specific human mAbs were cloned and expressed as described previously (Dejnirattisai et al., 2021a). Briefly, genes for Ig VH, Ig Vκ and Ig Vλ were recovered from positive wells by RT-PCR. Genes encoding Ig VH, Ig Vκ and Ig Vλ were then amplified using Nested-PCR by a cocktail of primers specific to human IgG. PCR products of HC and LCs were ligated into the expression vectors of human IgG1 or immunoglobulin κ-chain or λ-chain by Gibson assembly (Gibson, 2011). For mAb expression, plasmids encoding HCs and LCs were co-transfected by PEI-transfection into a HEK293T cell line, and supernatants containing mAbs were collected and filtered 4-5 days after transfection, and the supernatants were further characterized or purified.

#### Construction of Fab Expression Plasmids

Heavy chain expressing the specific mAbs were used as templates to amplify the heavy chain vector including the variable region and CH1 by Fab primers (Forward 5’- CAAGAGAGTTGAGCCCAAATCTTGTCTGGTGCCACGCGGAAGTAGTGCCTGGTCCCAC-3’, Reverse 5’- GTGGGACCAGGCACTACTTCCGCGTGGCACCAGACAAGATTTGGGCTCAACTCTCTTG-3’). The fragment with thrombin cleavage site and twin-strep tag overlapping with the Fab fragment were also amplified (Forward 5’-CATCCACAGTTCGAGAAATAGGTGCGACGGCCGGCAAG-3’, Reverse 5’- CTTGCCGGCCGTCGCACCTATTTCTCGAACTGTGGATG-3’). Fab fragment and tag fragment were joined by Gibson assembly (Gibson, 2011) and full plasmids were sequenced.

#### IgG mAbs and Fab Purification

To purify full length IgG mAbs, supernatants of mAb expression were collected and filtered by a vacuum filter system and loaded on protein A/G beads over night at 4 °C. Beads were washed with PBS three times and 0.1 M glycine pH2.7 was used to elute IgG. The eluate was neutralized with Tris-HCl pH8 buffer to make the final pH=7. The IgG concentration was determined by spectro-photometry and buffered exchanged into PBS.

To express and purify Fab, Fab heavy chain and light chain expression plasmids were co-transfected into HEK293T cells by PEI in Free-style 293 medium. After culturing for 5 days at 37°C with 5% CO2, culture supernatant was harvested and filtered using a 0.22 mm polyethersulfone filter. Fab were purified using the Strep-Tactin XT purification system.

#### Preparation of Fabs from IgGs

Fab fragments were digested from purified IgGs with papain using a Pierce Fab Preparation Kit (Thermo Fisher), following the manufacturer’s protocol.

#### Determination of mAb Binding to Recombinant *S*, RBD or NTD by ELISA

For spike, MAXISORP immunoplates (442404; NUNC) were coated with 2.5 μg/ml of StrepMAB-Classic (2-1597-001; iba) diluted by carbonate-bicarbonate buffer at 4°C overnight. Plates were blocked with 2% BSA dissolved by PBS for 1hr, and then 50 μl of 5 μg/ml of dual Strep-tagged S was added to each well and incubated for 1 hr at room temperate. 50 μl of mAb-expression supernatant or a series of dilutions of mAb was added, followed by ALP-conjugated anti-human IgG (A9544; Sigma) at 1:10,000 dilution. Plates were developed by adding PNPP substrate. After 40 min, the absorbance was measured at 405 nm.

To determine the binding to recombinant RBD or NTD, MAXISORP immunoplates were coated with 5 μg/ml of purified recombinant RBD-K417N, E484K, N501Y or NTD at 4 °C overnight. Plates were blocked with 2 % BSA dissolved by PBS for 1 h. After the addition of 50μl of mAb-expression supernatants or a series of dilutions of mAb, the rest of the procedure is the same as the S binding assay.

#### Focus Reduction Neutralization (FRNT)

The Focus reduction neutralization test was performed as previously described (Liu et al., 2021). Briefly, serially diluted Ab was mixed with SARS-CoV-2 strains Victoria, Alpha, Beta, Gamma, Alpha+E484K, Delta, or B.1.525 and incubated for 1 hr at 37 °C. The mixtures were transferred to 96-well, cell culture microplates containing confluent Vero cell monolayers in duplicate and incubated for 2 hr, followed by the addition of 1.5 % semi-solid carboxymethyl cellulose (CMC) overlay medium. A focus forming assay was then performed by staining Vero cells with human anti-NP mAb (mAb206) as primary antibody and peroxidase-conjugated goat anti-human IgG (A0170; Sigma) as secondary antibody. Finally, TrueBlue Peroxidase Substrate was added to each well to visualise the foci (infected cells). Virus-infected cell foci were counted on the classic AID EliSpot reader using AID ELISpot software.

### Quantification and statistical analysis

Statistical analyses are reported in the results and figure legends. Neutralization was measured by FRNT. The percentage of focus reduction was calculated and IC_50_ (FRNT50) was determined using the probit program from the SPSS package. The Wilcoxon matched-pairs signed rank test was used for the analysis and two-tailed P values were calculated on geometric mean values. BLI data were analysed using Data Analysis HT 11.1 (Fortebio) with a 1:1 fitting model.

#### ACE2 Binding Inhibition Assay by ELISA

MAXISORP immunoplates were coated with 5 μg/ml of purified ACE2-His protein overnight at 4 °C and then blocked by 2% BSA in PBS. Meanwhile, mAbs were serially diluted and mixed with 2.5 μg/ml of recombinant Beta trimeric S-twin-Strep. Antibody-S protein mixtures were incubated at 37°C for 1 hr. After incubation, the mixtures were transferred into the ACE2-coated plates and incubated for 1 hr at 37 °C. After wash, StrepMAB-Classic (2-1507-001, iba) was diluted at 0.2 μg/ml by 2% BSA and used as primary antibody followed by Goat anti-mouse IgG-AP (#A16093, Invitrogen) at 1:2000 dilution. The reaction was developed by adding PNPP substrate and stopped with NaOH. The absorbance was measured at 405nm. The ACE2/S binding inhibition was calculated by comparing to the antibody-free control well. IC50 was determined using the Probit program from the SPSS package.

#### Measurement of Viral Burden

Tissues were weighed and homogenized with zirconia beads in a MagNA Lyser instrument (Roche Life Science) in 1,000 μL of DMEM media supplemented with 2% heat-inactivated FBS. Tissue homogenates were clarified by centrifugation at 10,000 rpm for 5 min and stored at −80°C. RNA was extracted using the MagMax mirVana Total RNA isolation kit (Thermo Scientific) on a Kingfisher Flex extraction robot (Thermo Scientific). RNA was reverse transcribed and amplified using the TaqMan RNA-to-CT 1-Step Kit (ThermoFisher). Reverse transcription was carried out at 48°C for 15 min followed by 2 min at 95°C. Amplification was accomplished over 50 cycles as follows: 95°C for 15 s and 60°C for 1 min. Copies of SARS-CoV-2 N gene RNA in samples were determined using a previously published assay (Case et al., 2020; Hassan et al., 2020). Briefly, a TaqMan assay was designed to target a highly conserved region of the N gene (Forward primer: ATGCTGCAATCGTGCTACAA; Reverse primer: GACTGCCGCCTCTGCTC; Probe: /56-FAM/TCAAGGAAC/ZEN/AACATTGCCAA/3IABkFQ/). This region was included in an RNA standard to allow for copy number determination down to 10 copies per reaction. The reaction mixture contained final concentrations of primers and probe of 500 and 100 nM, respectively.

Statistical significance was assigned when *P* values were <0.05 using Prism Version 8 (GraphPad). Tests, number of animals, median values, and statistical comparison groups are indicated in each of the Figure legends. Analysis of weight change was determined by ANOVA https://www.socscistatistics.com/tests/anova/default2.aspx. Changes in viral burden were compared to control antibody-treated animals and analysed by one-way ANOVA with multiple comparisons tests.

#### Antibody Correlation Analysis

Each antibody was assigned a vector containing IC50 responses against seven virus strains (Victoria, Alpha, Beta, Gamma, Delta, B.1.525, Alpha+E484K) where the responses within each virus strain were normalised using a mean of 0 and a standard deviation of 1. Therefore, each scalar represented the deviation of an antibody’s IC50 response against a virus strain with respect to the average for that virus strain. These vectors were passed into cluster4x to generate correlation pairs for each antibody pair, as previously described for crystallographic datasets (Dejnirattisai et al., 2021a). The two largest modes of variation according to singular value decomposition were plotted. mAbs close to each other have similar behaviour in regards to neutralization against individual virus strains. Figures drawn using cluster4x (Ginn, 2020).

#### Bio-Layer Interferometry

BLI experiments were run on an Octet Red 96e machine (Fortebio). Competition assays of anti-Beta RBD mAbs were performed with Fortebio Ni-NTA Biosensors. His-tagged Beta RBD dissolved in the running buffer (10 mM HEPES, pH 7.4 and 150 mM NaCl) was used as the ligand and was first immobilized onto the biosensors. The biosensors were then washed with the running buffer to remove unbound RBD. Each biosensor was dipped into different saturating mAbs (Ab1) to saturate the bound RBD, except one biosensor was dipped into running buffer in this step, acting as the reference. Then all biosensors were washed with the running buffer again and dipped into wells containing the same competing antibody (Ab2). The y axis values of signals of different saturating antibodies in this step were divided by the value of the reference channel to get ratio results of different Ab1-Ab2 pairs. Ratio results close to 0 indicated total competition while 1 indicated no competition.

To measure the binding affinity of mAbs with Beta RBD, RBD was immobilized onto AR2G biosensors (Fortebio) and mAbs were used as analytes. All experiments were run at 30 °C. Data were recorded using software Data Acquisition 11.1 (Fortebio) and Data Analysis HT 11.1 (Fortebio) with a 1:1 fitting model used for the analysis.

#### Antibody Mapping Based on Bio-Layer Interferometry Competition Data

The procedure used the program Mabscape, described previously (Dejnirattisai et al., 2021a). In brief: competition values were prepared by capping all competition values between 0 and 1. Competition values between mAbs *i* and *j* were averaged with the competition value for *j* and *i* when both were available. A surface of the receptor-binding domain was generated in PyMOL (The PyMOL Molecular Graphics System, Version 1.2r3pre, Schrödinger, LLC) from chain E of PDB code 6YLA. A mesh was generated and iteratively contracted and restrained to the surface of the RBD to provide a smoother surface in Mabscape. A fixed position for those mAbs of known structure were objectively calculated from the atomic coordinates and locked to the nearest vertex on the mesh (FD5D (unpublished), EY6A (Zhou et al., 2020), S309 (Pinto et al., 2020) and mAbs 45, 75, 150 and 253) as previously described (Dejnirattisai et al., 2021a). In addition, mAbs 55, 58 and 61, which were given predicted locations from the previous study, were fixed at these predicted locations to aid mapping of the Beta mAbs. Beta mAbs which were of known structure were also locked according to the atomic coordinates (SA06, SA22, SA24, SA27, SA44, SA47, SA53, SA54). The target function was the sum of squared differences between the competition estimation and the competition value from SPR data. Minimisation was carried out globally by 1000 macrocycles using LBFGS refinement. Starting positions for mAbs were generated by randomly assigning a starting vertex on the RBD mesh and the target function minimised for 20 cycles considering data points for pairs with at least one fixed antibody, followed by 40 cycles for all data points. Between each cycle, antibody positions were locked onto the nearest mesh vertex. The average position for each antibody was chosen as the sampled position which had the lowest average square distance to every other sampled position, and the RMSD calculated from all contributing antibody positions (Dejnirattisai et al., 2021a; Ginn, 2020).

#### Crystallization

Endoglycosidase H1 was added to the purified Beta NTD and Beta RBD to remove glycan. Beta NTD was mixed with Beta-43 Fab, and Beta RBD was mixed separately with Beta-22, 24, 27, 38, 40 and 47 Fabs in a 1:1 molar ratio, with a final concentration of 13.0 mg ml^-1^. Beta RBD was combined with Beta-6 and COVOX-45 Fabs, Beta-49 and FI3A (PDB:7Q0A) Fabs, Beta-53 and Beta-29 Fabs, Beta-54 and Beta-37 Fabs, Beta-54 and Beta-44 Fabs and Beta-54 and Beta-50 Fabs in a 1:1:1 molar ratio all with a final concentration of 7 mg ml^−1^, separately. These complexes were separately incubated at room temperature for 30 min. Beta-32 Fab with a concentration of 35 mg ml^−1^ was also used for crystallization. Initial screening of crystals was set up in Crystalquick 96-well X plates (Greiner Bio-One) with a Cartesian Robot using the nanoliter sitting-drop vapor-diffusion method, with 100 nL of protein plus 100 nL of reservoir in each drop, as previously described (Walter et al., 2003). Crystals of Beta-NTD/Beta-43 Fab complex were formed in Molecular Dimensions Proplex condition 1-11, containing 0.1 M HEPES pH 7.5 and 25% (w/v) PEG 2000 MME. Crystals of Beta-RBD/Beta-22 Fab complex were formed in Molecular Dimensions Proplex condition 1-28, containing 0.2 M Lithium sulfate, 0.1 M MES pH 6.0 and 20% (w/v) PEG 4000. Crystals of Beta-RBD/Beta-24 Fab complex were formed in Proplex condition 1-40, containing 0.2 M Ammonium sulfate, 0.1 M Tris pH 7.5 and 20% (w/v) PEG 5000 MME. Crystals of Beta-RBD/Beta-27 Fab complex were formed in Proplex condition 1-36, containing 0.2 M Potassium iodide, 0.1 M MES pH 6.5 and 25% (w/v) PEG 4000. Crystals of Beta-RBD/Beta-38 Fab complex were formed in Proplex condition 2-32, containing 0.8 M Sodium/potassium phosphate pH 7.5. Crystals of Beta RBD/Beta-40 Fab complex were formed in Molecular Dimensions Morpheus condition 2-28, containing 12.5% (w/v) PEG 1000, 12.5% (w/v) PEG 3350, 12.5% (v/v) MPD, 0.02 M of each carboxylic acid and 0.1 M MES/imidazole pH 6.5. Crystals of Beta RBD/Beta-47 Fab complex were formed in Proplex condition 1-10, containing 0.1 M Potassium chloride, 0.1 M Tris pH 8.0 and 15% (w/v) PEG 2000 MME. Crystals of Beta RBD/Beta-6/COVOX-45 complex were formed in Hampton Research PEGRx condition 1-46, containing 0.1 M Sodium citrate tribasic dihydrate pH 5.0, and 18% (w/v) PEG 20000. Crystals of Beta RBD/Beta-49/FI3A and Beta RBD/Beta-54/Beta-50 complexes were formed in PEGRx condition 1-33, containing 0.1 M Tris, pH 8.0 and 28% (w/v) PEG 4000. Crystals of Beta RBD/Beta-53/Beta-29 complex were formed in Hampton Research Index condition 30, containing 0.1 M Sodium chloride, 0.1 M BIS-TRIS pH 6.5 and 1.5 M Ammonium sulfate. Crystals of Beta RBD/Beta-54/Beta-37 complex were formed in PEGRx condition 2-35, containing 0.15 M Lithium sulfate monohydrate, 0.1 M Citric acid pH 3.5 and 18% (w/v) PEG 6000. Crystals of Beta RBD/Beta-54/Beta-44 complex were formed in PEGRx condition 1-28, containing 0.1 M Citric acid pH 3.5 and 25% (w/v) PEG 3350. Crystals of Beta-32 Fab were formed in Index condition 23, containing 2.1 M DL-Malic acid pH 7.0.

#### X-ray Data Collection, Structure Determination and Refinement

Crystals were mounted in loops and dipped in solution containing 25% glycerol and 75% mother liquor for a second before being frozen in liquid nitrogen. Diffraction data were collected at 100 K at beamline I03 of Diamond Light Source, UK. All data were collected as part of an automated queue system allowing unattended automated data collection (https://www.diamond.ac.uk/Instruments/Mx/I03/I03-Manual/Unattended-Data-Collections.html). Diffraction images of 0.1° rotation were recorded on an Eiger2 XE 16M detector (exposure time from 0.004 to 0.01 s per image, beam size 80×20 μm, 30% beam transmission and wavelength of 0.9763 Å). Data were indexed, integrated and scaled with the automated data processing program Xia2-dials (Winter, 2010; Winter et al., 2018). 360° of data was collected from each crystal. Data set of Beta-32 Fab was merged from 4 crystals, data sets for Beta RBD complexes with Beta-22 and Beta-24 each was merged from 3 crystals, Beta-27 and Beta-29-Beta-53 each from 2 crystals, and the rest each from a single crystal.

Structures were determined by molecular replacement with PHASER (McCoy et al., 2007). The Beta RBD of the SARS-CoV-2 Beta RBD-EY6A-222 complex (PDB ID 7NXA) (Dejnirattisai et al., 2021b; Zhou et al., 2020), VhVl and ChCl domains which have the most sequence similarity to previously determined SARS-CoV-2 RBD/Fab structures (Dejnirattisai et al., 2021a, 2021b; Huo et al., 2020; Liu et al., 2021; Supasa et al., 2021; Zhou et al., 2020, 2021) were used as search models for each of the current structure determination. Model rebuilding with COOT (Emsley et al., 2010) and refinement with Phenix (Liebschner et al., 2019) were used for all the structures. Data collection and structure refinement statistics are given in Table S2. Structural comparisons used SHP (Stuart et al., 1979), residues forming the RBD/Fab interface were identified with PISA (Krissinel and Henrick, 2007) and figures were prepared with PyMOL (The PyMOL Molecular Graphics System, Version 1.2r3pre, Schrödinger, LLC) and UCSF ChimeraX (Pettersen et al., 2021)

#### Cryo-EM Grid Preparation

A 3 μL aliquot of S ∼1.2 μm with fab (1:6 molar ratio) was prepared, aspirated and almost immediately applied to a freshly glow-discharged Auflat 2/2-200 mesh holey grid (Protochips, supplied by Molecular Dimensions in the case of mAb 222) or C-flat 200 mesh 2/1 grid in the case of the remaining fab-S complexes at high intensity, 20 s, Plasma Cleaner PDC-002-CE, Harrick Plasma. Excess liquid was removed by blotting for 5 s with a force of -1 using vitrobot filter paper (grade 595, Ted Pella Inc.) at 4.5 °C, 100 % reported humidity before plunge freezing into liquid ethane using a Vitrobot Mark IV (Thermo Fisher). Fab/Spike complexes were incubated for 10-30 minutes prior to application to grids and plunge freezing.

#### Cryo-EM Data Collection

##### Beta S/Beta-44 Fab and Beta S/mAb 222 Fab

Data were collected in EER format using EPU on a 300 kV Titan Krios microscope equipped with a Falcon-IV detector with selectris energy filter. For Beta-44, a 50 μm aperture and a 100 μm objective were employed. A total of 20 K movies were recorded with a total dose of 45 e/Å^2^ with a pixel size 0.5 Å/pix with fringe free illumination in EER format.

##### Beta S/Beta-6, 26, 32, 43, 49, 50, 53

Compressed tif format movies were acquired on a Titan Krios (Thermo Fisher) operating at 300 kV with a K2 detector with 20 eV slit (Gatan) at a nominal magnification of 165 kX (corresponding to a calibrated pixel size of 0.82 Å/pix) and defocus range of 0.8-2.6 μm. For Beta-26, Beta-32, Beta-43, Beta-49, Beta-50 and Beta-53, movies were acquired also on a Titan Krios (Thermo Fisher) as for Beta-6 except with a 10 eV slit and 70 μm C2 aperture.

#### CryoEM Data Analysis

For all datasets, collected movies were 4-times binned and motion and ctf corrected on the fly using the Cryosparc live framework (Punjani et al., 2017). In most cases, particles were initially picked with the blob-picker module before spike-like particles from 2D classification of this initial set were used as a template for template-based picking.

##### Beta S with mAb 222 Fab

Two-times binned movies were motion corrected and aligned on the fly using Relion(3.1) scheduler (Zivanov et al., 2018) with a 5 x 5 patch based alignment. Aligned movies were then processed in Cryosparc (Punjani et al., 2017). For CoVOx-222, strong agreement between the fab/RBD position and that of the higher resolution crystal structure was observed. The majority of particles were in the ‘2-up’ configuration, with both upwards RBDs engaged with 222 fab. Given the superior resolution of the crystallographic structure of the RBD/222 complex, atomic-level detail was assessed with this structure instead. To generate an initial model, the deposited cryo-EM Beta 2-RBD up spike model (PDB: 7lyk [Gobeil et al., 2021]) was rigid body fitted into the locally filtered map in chimera (Pettersen et al., 2004) before the crystal structure of N501Y-mAb 222 (PDB: 7nx9) was initially superimposed on the upwards RBDs before rigid body fitting into the map (Liebschner et al., 2019). The Beta RBD-only model from crystal structure (residues 334-515) with Beta-22 was then aligned with the N501Y-mAb-222 RBD structure and combined with the mAb 222 fab. This RBD/fab model was then rigid body fitted (Liebschner et al., 2019) into the map before merging with the spike model.

##### Beta S with Beta-6

Using the Nu refine module of Cryosparc (Punjani et al., 2017), a total of 79905 particles from 6910 micrographs were used in the final 3D reconstruction, to 4.6 Å resolution (FSC = 0.143, Cryosparc) with an estimated b-factor of -175.5. To further resolve the interface between the spike/Fab, and between adjacent Fabs, additional local variability and local refinement processing were run. In addition, particles were exported into Relion (Zivanov et al., 2018) for 3D classification without alignment, with a mask around the Fab/RBD region. The resulting five classes, in agreement with 3D variability analysis, showed a small amount of movement between Fab/RBDs. For the full spike map, a model was created by combining our crystal structure with that of the Beta 2-up spike (PDB: 7lyk [Gobeil et al., 2021]), each rigid body fitted into the locally filtered cryo-EM map in Chimera (Pettersen et al., 2004). These models were then combined in coot before a further rigid body fit through Phenix (Liebschner et al., 2019). A good agreement was found between the crystal structure of Beta S only and Beta-6 Fab and a locally refined map focussing on the Fab-decorated RBD down and adjacent, also decorated, RBD up region, excepting the region between 472-492 for which a slight repacking of this loop was observed for both Fabs. However, the map quality was too poor in this region to reliably remodel the apparent slight twist in the loop in this region (especially since the fab CDR loops are not well modelled), even after deepEMhancer (Sanchez-Garcia et al., 2021) treatment of the map (Table S4). In addition to the contacts described between RBD plus Beta-6 inferred from the crystallographic structural analysis, residue S202/203 of the constant heavy domain of fab-decorating RBD-down appears to ‘kiss’ residues S66/G67 of the adjacent heavy variable domain. This interaction is, of course, an artifact of the relatively small size of a fab relative to a full antibody but could be a potential site for a cross-linked fab, whereby a S->C mutation at these two sites could be inserted.

##### Beta S with Beta-26

Particles were first picked with the blob-picker module within the Cryosparc framework (Punjani et al., 2017) before template picking, where a total of 224,609 particles were picked. Exposures were then further curated and picked particles classified twice, resulting in a set of particles with clear ‘antler’-like extensions consistent with Fab decorated spike. Ab initio followed by heterogeneous refinement into three classes yielded one class containing 50,878 particles with intact Fab-decorated spike. The final classified particle set refined to 4.04 Å reported resolution (-101.1 b-factor) with C1 symmetry, and 3.63 Å (-110.3 b-factor) with C3 symmetry. A clear 3-up spike configuration could be seen, with RBDs arranged in a similar ‘straight up’ position to that of anti-Victoria mAb 88 (Dejnirattisai et al., 2021a), with density commensurate with fab variable domain at the neck-left shoulder RBD region. For both the C1 and C3 symmetric maps, the Fab density was clear at low contour levels, but two Fabs were better defined than the third when no symmetry was imposed and appeared to be contacting the N-terminal region of the heavy chains. The ‘lone’ RBD-fab sits further back, away from its neighbours and twisted away from the nearest N-terminal domain. This was consistent with cluster analysis, where clusters showed two Fabs to lie closer together than the third. To better resolve the RBD/Fab interface, local refinements were performed with the two kissing Fabs, intersecting NTD and associated RBDs and also on one Fab/RBD/NTD alone (the procedure of mask generation for this is described in the Beta-32 sub-section below). Although somewhat improving the quality of the fab/RBD interface (see supplemental information), this was still insufficient to model and assess key interactions between the RBD and CDR loops.

##### Beta-S with Beta-32

A total of 856192 particles were picked using templates from blob picks as before from a total of 8177 aligned movies (Punjani et al., 2017). Particles were then filtered by 2d before 3d classification into 4 classes (using an ab initio model) resulting in a subset of 54,932 spike-like particles supporting clear fab decoration. Difficulties were encountered aligning this particle set, potentially due to the strong fab signal, and an initial non-uniform refinement was run (to 5.2 Å resolution) which was then used to run a focussed refinement on the more ordered inner portion of the spike (4.5 Å), which was then used as a basis for subsequent local variability analysis and local refinements. Further global classification with and without ab initio models failed to tease apart individual spike populations. For Beta-32, a blast search (Altschul et al., 1990) was conducted for the H and L chains and initial models were selected based on the sequence coverage, especially for the loop regions. For the H chain, 5U15 was found to be the most appropriate, with a single tyrosine substitution occurring at residue 114. For initial masking, this was curtailed to residues 1-130 H and 1-113 L for the two Fabs engaged with RBD in the up position (much variability was observed for the final fab). To generate a mask for local refinement, the Chimera colour zone module was used to extract the region of interest for masking from the map and was set to a radius of 15 Å so as to cover two Fab variable domains and associated RBDs, the NTD at the intersection between the two Fabs and the tip of the central helical bundle. This extracted region was then gaussian filtered and normalised to a mask within Cryosparc. An initial round of local refinement with a 5 Å and 5^o^ shift and angular search, masking this region from subtracted density, yielded a better albeit still low-resolution map at the Fab/RBD interface (reported resolution 6.9 Å, AuFSC = 0.143, as determined by Cryosparc [Punjani et al., 2017]). Local variability analysis of this map was then run to determine potential flexible regions that may be compromising the attained resolution. The crystal structure of Beta-32 was then rigid body fitted into the local map in chimera (Pettersen et al., 2021) and then coot before a single round of rigid body refinement in Phenix (Liebschner et al., 2019). One RBD appears to interact with the edge of the variable domain of the Fab decorating a neighbouring RBD.

##### Beta-S with Beta-43

Particles were picked with the blob-picker module from 13,982 movies within the Cryosparc live framework (Punjani et al., 2017) before this particle set was used as a template for the template picker module. Exposures were then further curated and picked particles classified, resulting in a set of 185,091 particles with clear ‘blade’-like extensions at the tips of the spike NTDs consistent with Fab decorated spike. Ab initio followed by heterogeneous refinement into three classes yielded one class containing 122,540 particles with Fab-decorated spike. Clusters from focussed 3D variable display analysis of these picked particles with a mask around the RBD/NTD/Fab region suggested the majority of Spike to have the RBD in the upwards position, but failed to convincingly separate different fab-decorated populations. Another set of three ab initio models followed by heterogenous classification was performed on this particle set and the resulting set of 90,286 particles were then unbinned yielding a final map to 4.85 Å (-209.2 b-factor) with C1 symmetry. A clear 1-up spike configuration could be seen.

##### Beta S with Beta-44

Particles were selected using the same procedure as before (418400 initially) before two rounds of 2D classification. These 149272 particles were then used to generate three ab initio models which were then used for 3d classification. Particles from a single class with clear decorated spike was then run through non-uniform refinement before a further ab initio model generation and classification into three classes. Again, only one class showed clear spike, and this final set of 61603 particles from 15710 movies was then extracted to the original box. Subsequent non-uniform refinement yielded a reconstruction to 3.9 Å resolution. However, despite extensive classification, density corresponding to Fab decorated RBD was poor. To better resolve the RBD/Fab interface, all of the spike except for one RBD and Fab was subtracted and focussed refinements were trialled with a fulcrum set to the RBD/Fab interface. However, the Fab signal was still very weak, likely due to a population of undecorated spike. To isolate a population of Fab decorated spike, local variability analysis in ‘cluster mode’ was employed. Two out of five clusters, corresponding to 21886 particles were found to be clearly decorated with Fab, with Fab signal being notably weaker for the final three. This final set of 21886 particles were locally refined, yielding a final low-resolution reconstruction of 8 Å resolution. After extensive classification, a species of spike, to 3.8 Å resolution, with two RBDs in the upwards condition, each decorated with weak Fab density was observed. The Fab/RBD interface was improved slightly after further classification and local refinement focussed on one Fab/RBD region. Since this was in agreement with the much higher-resolution crystal structure, it was not refined further.

##### Beta S with Beta-49

A total of 4523 movies were collected as described above. Particles, 854,545 were picked with the blob-picker module within the Cryosparc live framework (Punjani et al., 2017) while exposures were curated within the live interface as before. 2D classification resulted in the isolation of 312,924 particles with clear antler extensions. Three ab initio models were then used in heterogeneous refinement with C3 symmetry into three classes yielded one class containing 225,466 particles with intact Fab-decorated spike. This particle set was refined using the nu-refinement module in Cryosparc with both C1 and C3 symmetry before global and local CTF refinement followed by a second non-uniform refinement run and local filtering. The C3 symmetry map (2.55 Å reported res, -76 b-factor, versus 2.7 A, -68.4 b-factor) appear to be marginally worse at the RBD/fab interface and thus the C1 map was used for model building. Unlike most other reconstructions presented in this paper, the RBD-fab interface was well resolved, and this Spike was used as a basis for some of the other structure building.

##### Beta S with Beta-50

From 6,880 movies, particles were initially picked with the blob-picker module within the Cryosparc live framework (Punjani et al., 2017) before a set of ca. 9000 ‘good’ side views of decorated particles were used as a template for template picking on a set of curated exposures. 2D classification resulted in the isolation of 312,924 particles with clear antler extensions. Three ab initio models were then used in heterogeneous refinement with C3 symmetry into three classes yielded one class containing 225,466 particles with intact Fab-decorated spike. This particle set was refined using the nu-refinement module in Cryosparc with both C1 and C3 symmetry before global and local CTF refinement followed by a second non-uniform refinement run and local filtering. The C3 symmetry map (2.55 Å reported res, -76 b-factor, versus 2.7 Å, -68.4 b-factor) appear to be marginally worse at the RBD/fab interface and thus the C1 map was used for model building. The RBD-fab interface was well resolved.

##### Beta S with Beta-53

A significant population of Spike decorated with three Fabs was observed after preliminary processing. Three Fabs were found to decorate each of the three RBDs of the spike protein, and density for these was strong. Evidence for Beta-53 being disruptive was observed in 2D class averages, where a single RBD plus Fab was observed after an incubation of ca. 20 minutes. Comparison of spike distribution with an incubation in absence of antibody confirmed some Fab mediated disruption.

##### Alphafold

To generate a fab model for Beta-26, alphafold2 (Jumper et al., 2021) was employed. For this, two strategies were trialled. First, the Heavy and Light chain amino acid sequences were separately submitted and the resulting models independently rigid body fitted into the locally refined map. However, the map density was too poor for confident modelling and there were significant clashes at the H/L interface. Alignment of the H and L chains with the closest PDB sequence match led to an unsatisfactory fit in the fab density. The second strategy, which was more successful, involved submitting the H and L variable domain sequences together with a 19 residue Ser/Gly linker [SSSGGGGSGGGGSGGGGSS]. The linker length and content were decided based on a BLAST search with the H and L sequences together. The best fit out of the five alphafold2 models was then rigid body fitted into the density and the linker region removed. Some clashes remained in the model, and the map density was too poor to confidently resolve these.

##### Cryo-EM Model Refinement

Since the fab-RBD region was so resolved for many cryo-EM maps, models of RBD, fab and S without RBD were rigid body fitted into the final map for those reconstructions. For structures where local refined maps were available, the RBD-fab model was treated as a rigid body. In each case, the interface between RBD and remaining S model was checked in coot before a final round of rigid body refinement in Phenix whereby the entire S-fab complex model (or, in the case of local refinements, RBD-fab model) was treated as a rigid body. Beta-53, mAb-222, Beta-49, Beta-50 and Beta-44 S maps were globally good resolution, below 4 Å, except for much of the NTD, RBD and Fab components. In these cases, RBD/NTD-fab regions, for which the crystal structure was used, were treated as a rigid body, while ADP refinement was run using Phenix on the remaining portion of the model before further inspection in Coot.

## Data Availability

• Coordinates and structure factors of the crystallographic complexes have been deposited in the PDB and are publicly available as of the date of publication. Accession codes are listed in the key resources table. Coordinates and maps for the cryo-EM complexes (only the map for Beta-44) have been deposited in the PDB and are publicly available as of the date of publication. Accession codes are listed in the key resources table. • Any additional information required to re-analyze the data reported in this paper is available from the lead contact upon request.

## Supplemental information

Document S1. Figures S1–S5 and Tables S1–S4

Document S2. Article plus supplemental information

## Footnotes

Supplemental information can be found online at https://doi.org/10.1016/j.chom.2021.11.013.
