# Supplementary material for: The antibody response to SARS-CoV-2 Beta underscores the antigenic distance to other variants
Source: Cell Host Microbe. 2022 Jan 12;30(1):53–68.e12. doi: 10.1016/j.chom.2021.11.013 (PMC8626228; doi:10.1016/j.chom.2021.11.013)
Supplement: Document S1. Figures S1–S5 and Tables S1–S4 [file mmc1.pdf]

Cell Host & Microbe, Volume *30*

Supplemental information

The antibody response to SARS-CoV-2 Beta underscores the antigenic distance to other variants

Chang Liu, Daming Zhou, Rungtiwa Nutalai, Helen M.E. Duyvesteyn, Aekkachai Tuekprakhon, Helen M. Ginn, Wanwisa Dejnirattisai, Piyada Supasa, Alexander J. Mentzer, Beibei Wang, James Brett Case, Yuguang Zhao, Donal T. Skelly, Rita E. Chen, Sile Ann Johnson, Thomas G. Ritter, Chris Mason, Tariq Malik, Nigel Temperton, Neil G. Paterson, Mark A. Williams, David R. Hall, Daniel K. Clare, Andrew Howe, Philip J.R. Goulder, Elizabeth E. Fry, Michael S. Diamond, Juthathip Mongkolsapaya, Jingshan Ren, David I. Stuart, and Gavin R. Screaton

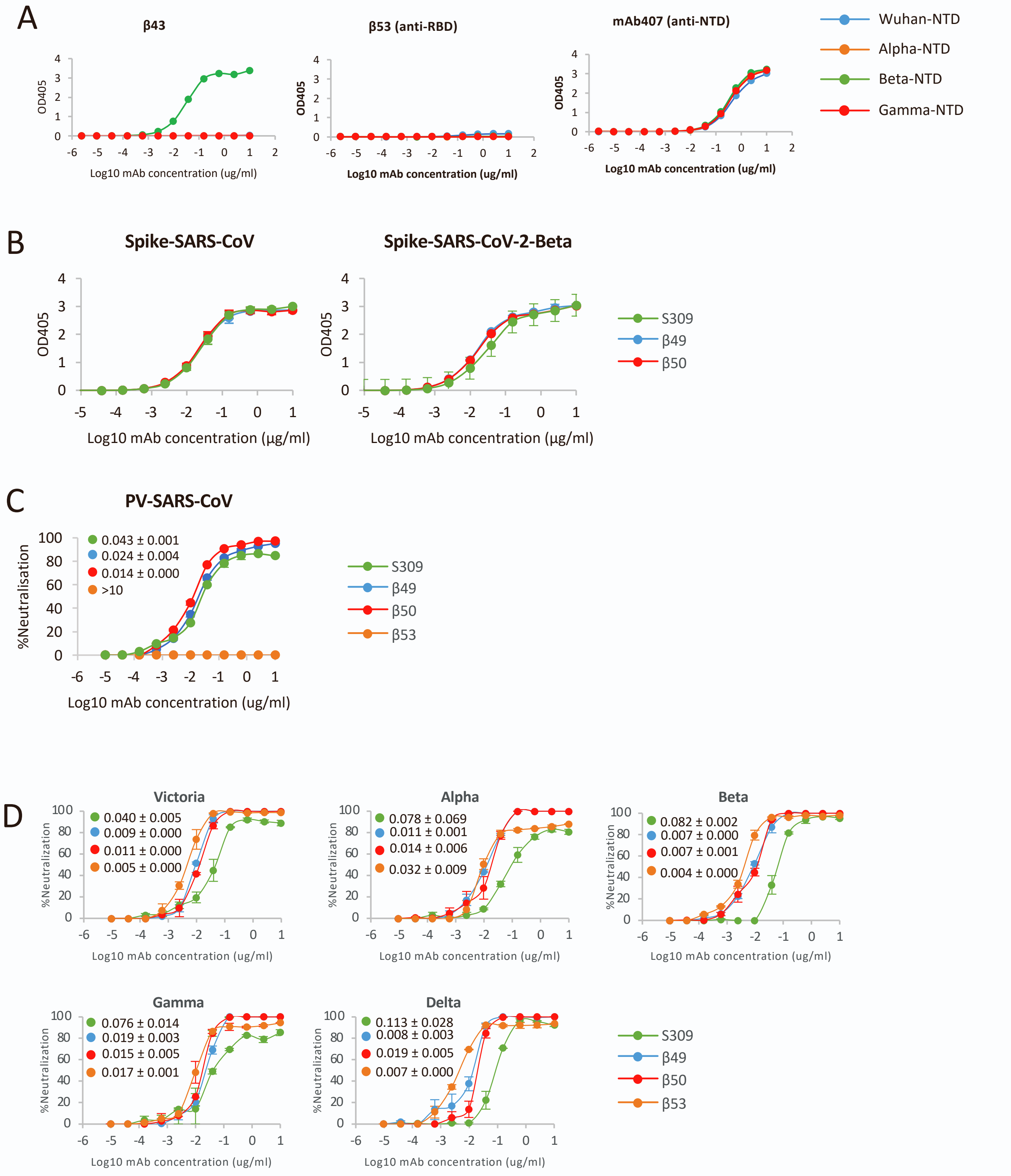

Figure S1. Binding / neutralisation comparisons of Beta-43 and Beta-49/50. (A) Binding of Beta-43 to plate immobilized Wuhan, Alpha, Beta and Gamma NTDs was determined by ELISA, mAb 407 is a cross reactive anti-NTD mAb. (B) Cross reactivity of Beta 49, 50 with SARS-CoV-1 S trimer measured by ELISA and compared with mAb S309. (C) SARS-CoV pseudovirus neutralisation curves for mAb S309, Beta-49, 50, 53. (D) Neutralization curves of Beta using mAb S309, Beta-49, 50, 53. The data points for S309 have been previously published (Dejnirattisai et al., 2021b). Related to Figure 2.

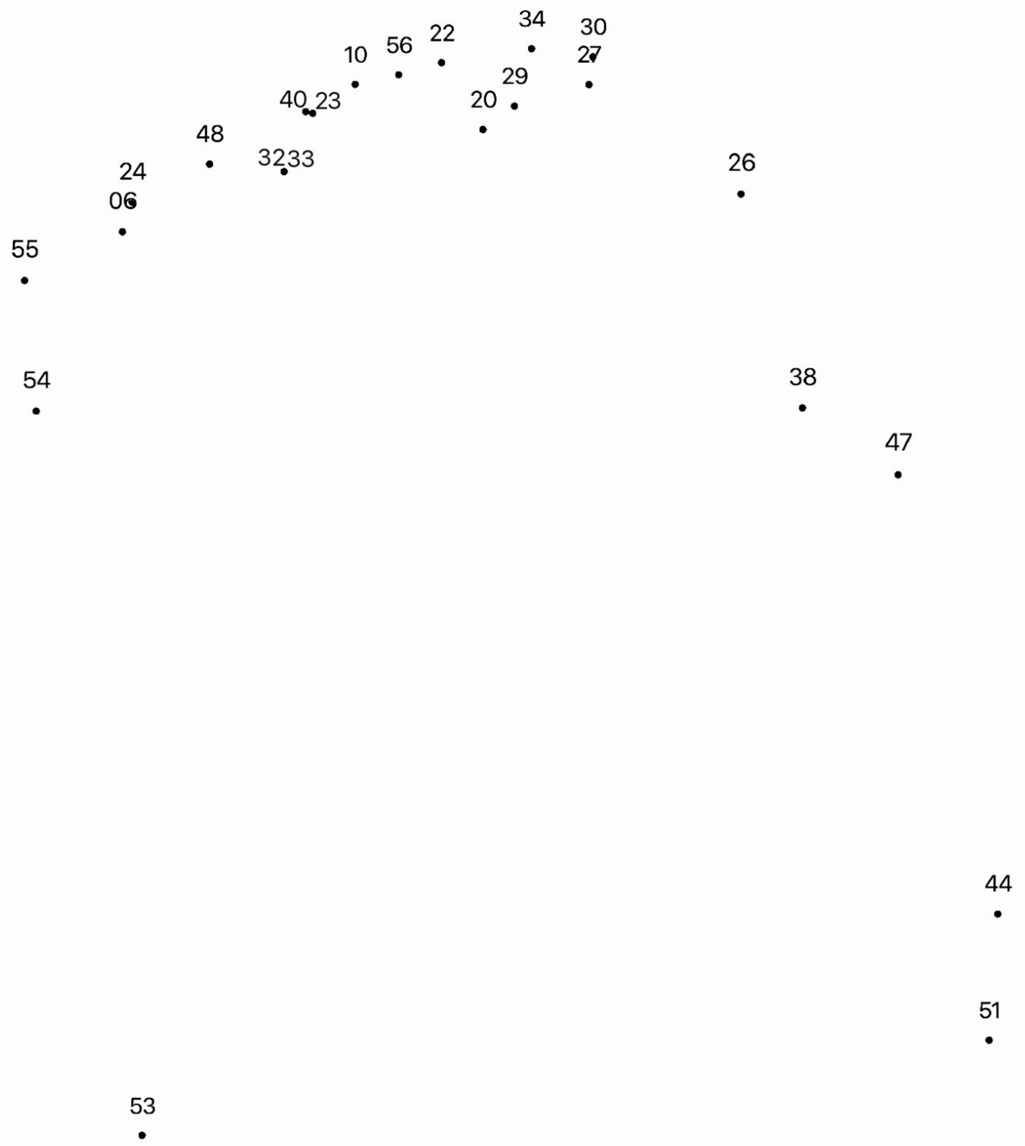

Figure S2. Cluster analysis (see Methods) of the pairwise BLI competition experiment. The points are projected onto two dimensions with the minimal information loss. There is a reasonably continuous distribution which essentially runs from one flank up across the shoulder, neck, shoulder to the opposite flank in the torso analogy. Antibody names are abbreviated, from Beta-n to simply n. Related to Figure 3.

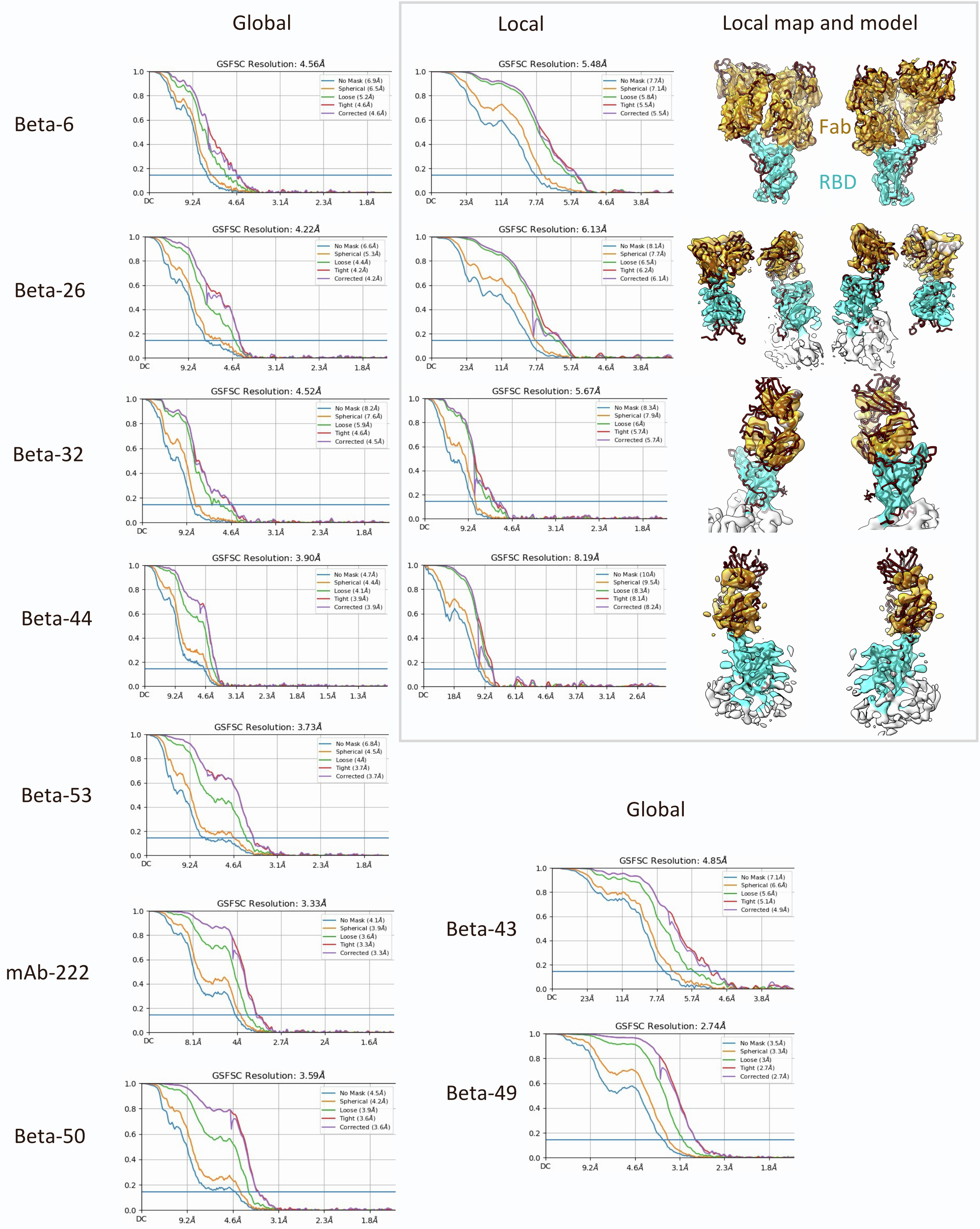

Figure S3. Gold-standard FSC curves for global and locally refined S-Fab complexes. Local refined maps are colored in cyan, orange and light grey for RBD, Fab and the rest respectively, and fitted RBD/Fab models in burgundy (with black outline). Map/model pairs are 180° rotated with respect to one another. Related to Figure 4.

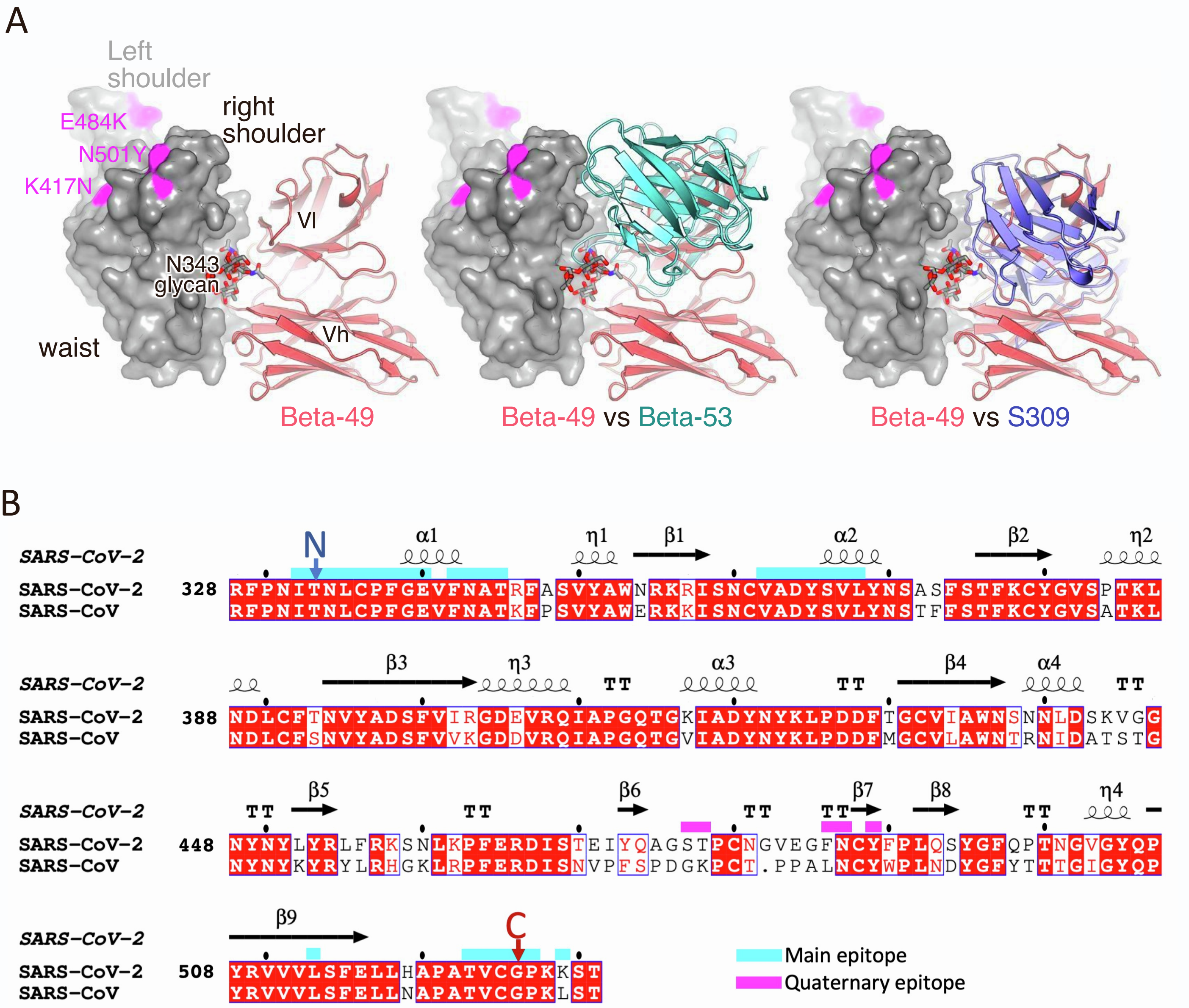

Figure S4. Epitopes of antibodies Beta-49 and 50. (A) The binding mode of Beta-49 compared to Beta-53 and S309. The crystal structure of Beta-49/Beta-RBD is shown with the RBD as grey surface with the three mutation sites highlighted in magenta, the VhVl as ribbons and N343 glycans as sticks (left panel). The middle panel compares the binding mode of Beta-49 to Beta-53 (cyan, PDB ID 7PS2), and the right panel to S309 (blue, PDB ID 7BEP). (B) Sequences of the RBD region for SARS-CoV-2 and SARS-CoV-1. The conserved major epitope is marked in cyan, N and C mark the ends of the natural sequence in the soluble RBD construct. Secondary structure in SARS-CoV-2 is marked above and sequence numbers are given for SARS-CoV-2. Drawn with ESPript (Gouet et al 1999). Related to Figure 6.

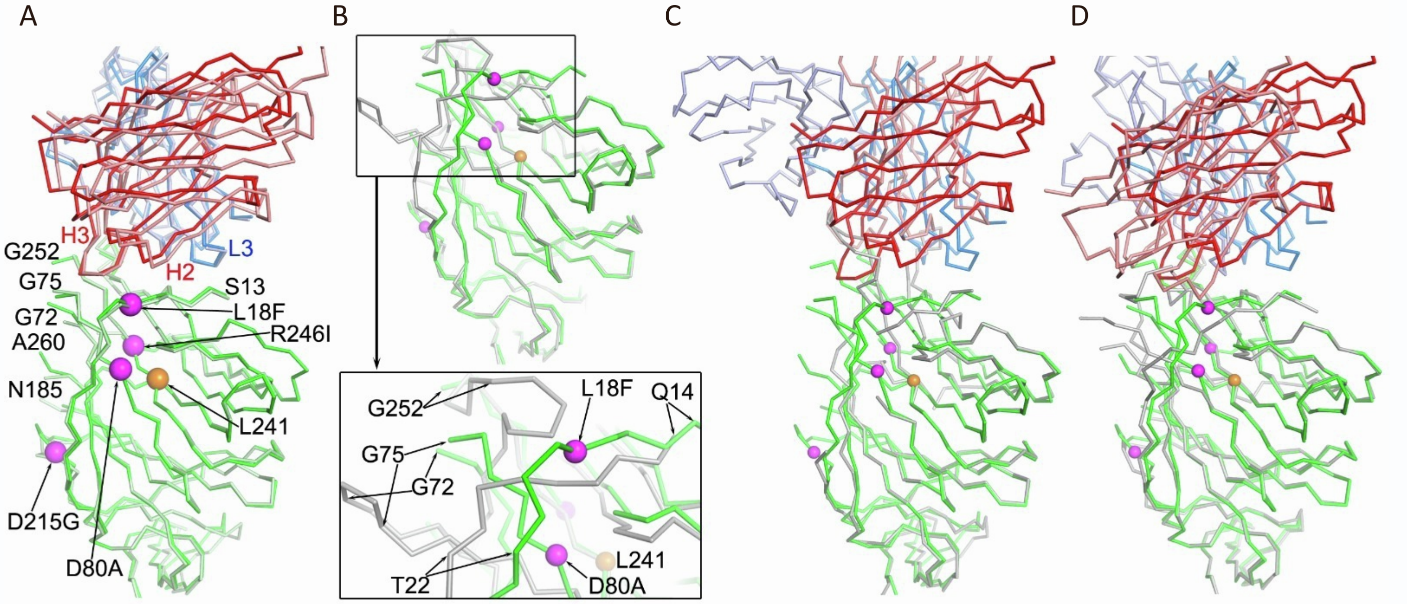

Figure S5. NTD conformational changes. Comparison of the crystal structures of Beta-43 bound Beta-NTD and that of the original virus strain. (A) Comparing the two Beta-NTD/Beta-43 complexes in the crystal asymmetric unit by overlapping the Beta-NTD. The Beta-NTD, HC and LC are coloured green, red and blue in one complex, and pale green, salmon and light blue in the second. Mutation sites are shown as magenta spheres. The Cα of residue 241 is in orange, after which there is a three-residue deletion in the Beta variant. (B) Overlap of the Beta-NTD (green) with the biliverdin bound NTD (Grey, PDB ID 7B62). The boxed area shows the large differences between the two structures. (C), (D) Comparisons of Beta-NTD/Beta-43 complex (coloured as in (A)) with NTD/N11 Fab complex (C) (grey, salmon and light blue for NTD, HC and LC respectively. PDB ID 7E7X) and NTD/S2M28 Fab complex (D) (colour scheme is as for (C). PDB ID 7LY3). Related to Figure 7.

Table S1A. IC50 titres of 28 Beta SARS-CoV-2-specific human mAbs against live virus strains Victoria, B.1.1.7, B.1.351, P.1, B.1.1.7+E484K, B.1.525 and 1.617.2. The data are shown as mean±s.e.m. of 2 independent experiments. Related to Figure2.

mAbs	IC50 (ug/ml)						
	Victoria	Alpha	Beta	Gamma	Alpha+E484K	B.1.525	Delta
β06	>10	0.024 ± 0.002	0.008 ± 0.002	0.015 ± 0.003	0.034 ± 0.003	>10	>10
β10	>10	0.064 ± 0.042	0.015 ± 0.000	0.025 ± 0.011	0.322 ± 0.071	>10	>10
β20	>10	>10	0.005 ± 0.001	0.345 ± 0.122	>10	>10	>10
β22	>10	6.58 ± 2.988	0.025 ± 0.004	0.030 ± 0.007	3.672 ± 1.538	>10	>10
β23	>10	0.009 ± 0.001	0.011 ± 0.001	0.020± 0.000	0.019 ± 0.002	>10	>10
β24	>10	0.007 ± 0.001	0.002 ± 0.001	0.005 ± 0.001	0.011 ± 0.003	>10	>10
β26	2.742 ± 0.208	>10	0.012 ± 0.003	0.016 ± 0.000	0.030 ± 0.009	0.025 ± 0.001	>10
β27	0.018 ± 0.002	0.018 ± 0.000	0.009 ± 0.000	0.006 ± 0.002	0.026 ± 0.002	0.025 ± 0.004	0.021 ± 0.004
β29	>10	1.372 ± 0.016	0.027 ± 0.003	0.023 ± 0.009	0.149 ± 0.065	>10	>10
β30	2.643 ± 0.88	0.004 ± 0.001	0.003 ± 0.001	0.004 ± 0.000	0.007 ± 0.002	0.966 ± 0.239	0.350 ± 0.035
β32	0.248 ± 0.003	0.119 ± 0.044	0.053 ± 0.025	0.027 ± 0.014	0.173 ± 0.097	0.257 ± 0.171	0.267 ± 0.068
β33	2.016 ± 0.051	0.234 ± 0.013	0.017 ± 0.003	0.017 ± 0.001	0.042 ± 0.014	1.359 ± 0.406	0.334 ± 0.005
β34	8.241 ± 1.067	1.466 ± 0.136	0.032 ± 0.010	0.092 ± 0.003	0.072 ± 0.002	0.387 ± 0.034	>10
β38	>10	>10	0.011 ± 0.003	0.043 ± 0.025	0.017 ± 0.010	0.021 ± 0.006	>10
β40	0.075 ± 0.005	0.001 ± 0.000	0.001 ± 0.000	0.001 ± 0.000	0.001 ± 0.001	0.118 ± 0.045	0.107 ± 0.031
β43	>10	>10	0.048±0.024	>10	>10	>10	>10
β44	0.007 ± 0.002	0.028 ± 0.008	0.015 ± 0.008	0.071 ± 0.026	0.035 ± 0.005	0.019 ± 0.001	>10
β45	>10	>10	0.018 ± 0.003	0.015 ± 0.006	0.068 ± 0.010	0.027 ± 0.005	>10
β47	0.006 ± 0.001	0.008 ± 0.003	0.003 ± 0.001	0.004 ± 0.002	0.018 ± 0.003	0.010 ± 0.007	0.005 ± 0.000
β48	0.034 ± 0.011	0.020 ± 0.008	0.009 ± 0.001	0.011 ± 0.001	0.011 ± 0.001	0.087 ± 0.016	0.042 ± 0.016
β49	0.009 ± 0.000	0.011 ± 0.001	0.007 ± 0.000	0.019 ± 0.003	0.014 ± 0.002	0.008 ± 0.002	0.008 ± 0.003
β50	0.011 ± 0.000	0.014 ± 0.006	0.007 ± 0.001	0.015 ± 0.005	0.010 ± 0.002	0.018 ± 0.001	0.019 ± 0.005
β51	0.119 ± 0.008	0.242 ± 0.024	0.005 ± 0.000	0.019 ± 0.001	0.008 ± 0.003	0.005 ± 0.001	>10
β53	0.005 ± 0.000	0.032 ± 0.009	0.004 ± 0.000	0.017 ± 0.001	0.023 ± 0.009	0.008 ± 0.001	0.007 ± 0.000
β54	0.232 ± 0.092	0.002 ± 0.001	0.001 ± 0.000	0.002 ± 0.001	0.002 ± 0.000	0.834 ± 0.247	0.409 ± 0.071
β55	0.108 ± 0.069	0.028 ± 0.004	0.01 0± 0.003	0.022 ± 0.001	0.014 ± 0.003	0.075 ± 0.016	0.076 ± 0.020
β56	0.046 ± 0.013	0.001 ± 0.000	0.001 ± 0.000	0.002 ± 0.000	0.002 ± 0.000	0.034 ± 0.014	0.022 ± 0.002

Table S1B. Immunoglobulin heavy and light chain gene families of 28 Beta SARS-CoV-2-specific human mAbs. Related to Figure 3

mAbs	Patient No.	Predicted contact residues	HC				LC			
			V-GENE	J-GENE	D-GENE	#Amino acid substitutions	K/λ	V-GENE	J-GENE	#Amino acid substitutions
β06	SA02	501Y	4-39*07	4*02	3-16*02	9	K	3-11*01	5*01	7
β10	SA02	501Y	4-39*07	5*02	3-16*01	5	K	1-9*01	5*01	2
β20	SA02	417N/T	3-33*08	6*03	5-18*01	7	K	3-20*01	4*01	3
β22	SA02	417N/T, 501Y	3-30*03 or 3-30*18 or 3-30-5*01	6*02	2-2*01	7	K	4-1*01	3*01	7
β23	SA02	501Y	4-39*07	4*02	3-10*01	5	λ	3-1*01	1*01	7
β24	SA02	501Y	4-30-4*08	5*02	2-2*01	8	λ	2-23*01 or 2-23*03	1*01	10
β26	SA04	484K, 452L/478T	4-59*01	3*02	2-15*01	8	λ	3-21*02	1*01	4
β27	SA04	Fully cross-neutralizing	3-53*04	6*02	3-3*01	7	K	3-20*01	4*01	7
β29	SA04	501Y, 417N/T	3-30*03 or 3-30*18 or 3-30-5*01	3*01	2-21*02	11	K	4-1*01	5*01	5
β30	SA15	501Y	1-2*02	5*02	4-11*01	8	K	1-17*01	3*01	4
β32	SA15	Fully cross-neutralizing	1-2*02	5*02	3-22*01	5	K	1-12*01 or 1-12*02 or 1D-12*02	1*01	1
β33	SA15	484K	1-2*02	6*02	3-16*01	6	λ	3-25*03	1*01	7
β34	SA02	484K, 452L/478T	3-48*04	3*02	1-14*01	7	K	3-11*01	4*01	5
β38	SA15	484K	5-51*03	6*02 or 6*03	1-26*01	4	λ	1-44*01	1*01	2
β40	SA15	501Y	4-39*01	5*02	2-15*01	6	λ	3-25*03	2*01 or 3*01	13
β43	SA15	Non-RBD	3-33*01 or 3-33*06	6*02	2-15*01	4	λ	3-21*04	1*01	6
β44	SA15	452L/478T	1-18*01	5*02	3-10*01	2	λ	2-23*01 or 2-23*03	3*02	3
β45	SA15	484K	3-23*04	6*02	2-2*01	15	K	1-33*01 F, or 1D-33*01	4*01	4
β47	SA18	Fully cross-neutralizing	1-58*03	3*02	2-2*01	9	K	3-20*01	3*01	6
β48	SA18	Fully cross-neutralizing	3-21*01	3*02	1-26*01	9	K	3-15*01	1*01	10
β49	SA18	Fully cross-neutralizing	1-69*01, or 1-69D*01	4*02	1-26*01	8	K	3-20*01	1*01	4
β50	SA18	Fully cross-neutralizing	1-69*01, or 1-69D*01	4*02	1-26*01	11	K	3-20*01	2*01	6
β51	SA18	484K, 452L/478T	1-24*01	4*02	6-19*01	5	K	1-16*02	5*01	3
β53	SA18	Fully cross-neutralizing	5-10-1*03	4*02	5-24*01	7	K	3-15*01	1*01	3
β54	SA18	501Y	4-39*01	5*02	6-19*01	10	K	1-9*01	4*01	0
β55	SA18	501Y	4-39*01 F	3*02	3-22*01	7	K	1-5*03	1*01	7
β56	SA18	501Y	4-31*06	4*02	6-6*01	12	λ	2-14*03	2*01 or 3*01	10

Table S2. X-ray data collection and structure refinement statistics. Related to Figure 4.

Structure	RBD/Beta-6-Fab45 *	RBD/Beta-22[a]	RBD/Beta-24	RBD/Beta-27	RBD/Beta-29-Beta-53 *	Beta-32 Fab	RBD/Beta-38	RBD/Beta-40	RBD/Beta-47[a] *	RBD/Beta-44-Beta-54	RBD/Beta-49-FI3A	RBD/Beta-50-beta-54	NTD/Beta-43
**PDB ID**	7PRY	7PRZ	7PS0	7PS1	7PS2	7PS3	7PS4	7PS7	7PS5	7PS6	7Q0G	7Q0H	7Q0I
**Data collection**													
Space group	$P2_1$	$R32$	$P2_1$	$C2$	$R32$	$P2_12_12_1$	$P2_1$	$P2_1$	$P2_12_12_1$	$P2_12_12$	$P2_12_12_1$	$P2_1$	$P2_1$
Cell dimensions													
a, b, c (Å)	86.4, 166.8, 120.2	199.4, 199.4, 202.4	81.8, 110.8, 85.4	193.4, 85.7, 57.4	213.4, 213.4, 226.8	60.6, 66.8, 135.0	55.1, 118.5,109.3	41.2, 139.9, 116.4	78.1, 114.4, 169.0	129.4, 112.6, 99.9	89.3, 106.5, 215.6	102.2, 42.5, 138.4	117.8, 67.0, 143.2
α, β, γ (°)	90, 108.5, 90	90, 90, 120	90, 102.7, 90	90, 99.7, 90	90, 90, 120	90, 90, 90	90, 93.5, 90	90, 100.0, 90	90, 90, 90	90, 90, 90	90, 90, 90	90, 99.9, 90	90, 92.5, 90
Resolution (Å)	74–3.10 (3.15–3.10)[b]	87–3.20 (3.25–3.20)	67–2.92 (2.97–2.92)	95–2.40 (2.44–2.40)	50–2.99 (3.04–2.99)	68–1.70 (1.73–1.70)	59–1.94 (1.97–1.94)	60–3.90 (3.96–3.90)	60–3.14 (3.20–3.14)	65–2.26 (2.30–2.26)	108–1.82 (2.05–1.82)	89–3.65 (3.71–3.65)	143–2.39 (2.58–2.39)
R_{merge}	0.246 (---)	0.473 (---)	0.482 (---)	0.181 (---)	0.293 (---)	0.181 (---)	0.065 (---)	0.389 (---)	0.229 (---)	0.096 (---)	0.095 (---)	0.550 (---)	0.144
R_{pim}	0.106 (0.981)	0.063 (0.897)	0.108 (0.812)	0.042 (0.501)	0.048 (0.885)	0.027 (1.887)	0.029 (0.966)	0.166 (1.435)	0.068 (1.472)	0.029 (1.435)	0.027 (0.403)	0.226 (1.146)	0.059 (0.577)
$I/\sigma(I)$	6.6 (0.5)	9.4 (0.5)	4.8 (0.6)	8.6 (0.4)	8.5 (0.3)	13.4 (0.5)	17.1 (0.4)	4.9 (0.5)	7.8 (0.3)	14.2 (0.3)	18.3 (1.9)	3.7 (0.5)	9.8 (1.4)
$CC_{1/2}$	0.984 (0.340)	0.998 (0.549)	0.988 (0.467)	0.995 (0.599)	0.998 (0.429)	0.999 (0.705)	0.990 (0.321)	0.988 (0.246)	0.998 (0.365)	0.974 (0.265)	0.999 (0.711)	0.950 (0.434)	0.997 (0.478)
Completeness (%)	100 (98.4)	99.8 (95.9)	99.7 (93.1)	96.7 (79.0)	99.9 (98.5)	99.9 (96.4)	98.0 (79.9)	99.4 (98.5)	100 (100)	99.5 (92.3)	94.9 (86.4)	100 (98.6)	93.6 (60.1)
Redundancy	6.4 (6.4)	57.5 (59.5)	20.9 (20.2)	16.7 (5.7)	38.4 (41.7)	43.4 (17.7)	6.2 (4.1)	6.3 (7.1)	12.2 (13.1)	12.3 (8.5)	13.8 (12.9)	6.8 (6.3)	6.9 (7.0)
**Refinement**													
Resolution (Å)	58–3.10	87–3.20	65–2.92	57–2.40	48–2.99	48–1.70	55–1.94	57–3.90	57–3.14	50–2.26	108–1.82	89–3.65	143–2.39
No. reflections	55715/2711	24332/1326	30862/1496	32810/1642	38058/2011	57383/3002	95822/5034	11260/568	25227/1307	63593/3455	89133/4708	12841/705	58316/2931
R_{work} / R_{free}	0.245/0.290	0.190/0.223	0.222/0.271	0.190/0.216	0.234/0.267	0.171/0.188	0.199/0.231	0.233/0.284	0.226/0.274	0.212/0.239	0.192/0.224	0.255/0.303	0.202/0.242
No. atoms													
Protein	16222	4954	9487	4767	8293	3387	9625	9460	4876	8032	8068	8142	11158
Ligand/ion/water	76	137	58	227	138	418	528	---	131	129	513	60	446
B factors (Å^2)													
Protein	107	130	75	55	146	38	67	172	128	80	47	126	52
Ligand/ion/water	143	189	110	58	153	46	59	---	193	86	45	114	69
r.m.s. deviations													
Bond lengths (Å)	0.003	0.003	0.002	0.002	0.003	0.004	0.006	0.008	0.003	0.003	0.008	0.003	0.003
Bond angles (°)	0.5	0.6	0.5	0.5	0.6	0.7	0.8	0.8	0.5	0.6	1.0	0.6	0.6

* In Beta-47/RBD, the RBD most distant from the epitope (close to the N- and C-terminal regions) is not well ordered. In Beta-29/Beta-53/RBD and Beta-6/Fab-45/RBD the constant domains of both Fabs are less well ordered.

[b] SA022 glycosylated at N35 of the light chain, and SA047 at N102 of the heavy chain.

[c] Values in parentheses are for highest-resolution shell.

Table S3. Sequences for heavy chain CDRs for IgVH4-30, 4-39, 3-30 and 3-33 gene families and for other structures determined in this paper. Related to Figure 5.

	CDR-H1	CDR-H2	CDR-H3
**IgVH4-30**	*	*	
Beta-24	CSVSDGSISSSDYYWSW	EWIGYIYYT-GSTYY	YYCARLVVPS-----PKGSWFDPWG
**IgVH4-39**			
Beta-6	CTVSGGSISSSSHYWGW	EWIGSIYYS-ESAYY	YYCARVTEPR-----WTSCYFDYWG
Beta-10#	CTVSGGSISSSSYYWGW	EWIGSIYYS-GSTYY	YYCARERSAP-----LAGNWFDPWG
Beta-23#	CTVSGGSISSSSYYWGW	EWIGNVYYS-GGTYC	YYCARIWFGE-----PAGGYFDYWG
Beta-40	CTVSGGSISSSSYYWGW	EWIGSIYYS-GSAYY	FYCARHAAPS-----PGDNWFDPWG
Beta-54	CTVFGGSITSSNHYWVW	EWIGSMYYS-GSTAY	YYCARQIGPKRPS--QVADWFDPWG
Beta-55#	CTVSGDSISSSRYYWGW	EWIGTFYYS-GITYY	YYCARPRPPDYYDN-SGALLFDIWG
**IgVH3-30**			
Beta-22	CAASG--FTFSNYGIHW	EWVAVISYDGSHKYY	YYCAKDSSAAIP---YYYYGMDVWG
Beta-29	CAASG--FTFSNYGMHR	EWVALISYEESNRYY	YYCAKDQGPATVMVTAIRGAMDVWG
**IgVH3-33**			
Beta-20#	CAASG--FPFSNYGMHW	EWVAVIWYDGSNKYY	YYCAKDGYTA----HYYYYYMDVWG
**IgVH1-69**			
Beta-49	CKASG--GTFSSSVISW	EWMGGIIPLFGSANY	YYCAKV---------SQWALILFWG
Beta-50	CKASR--GTFNTYVFTW	EWMGGIIPFFGTADY	YYCSRL---------SQWDLLPMWG
**Others with known structure**			
Beta-26	CTVSG--ASISNYYWSW	EWVGYIYYT-GSTNH	YYCARAYCSGG----SCFDTFDIWG
Beta-27	CAASG--LTVRSNYMNW	EWVSLIYS-GGSTFY	YYCARD---------LVVYGMDVWG
Beta-32	CKASG--YTFTGYYMHW	EWMGWINPNSGGTNY	YYCARVGAHDYYD--SSDNWFDPWG
Beta-38	CKGSG--YSFTNYWIGW	EWMGIIYPGDSGTRY	YYCARSRVGATGG--YYDYYMDVWG
Beta-43	CAASG--FTFSSYGMHW	EWVAVIWYDGSNNFY	YYCARSYCSGGFC-FGYYYGLDVWG
Beta-44	CKASG--YTFTSYGISW	EWMGWISPYNGNTHY	YYCARDGE--------LLGWFDPWG
Beta-47	CKASG--FTFITSAVQW	EWMGWIAVGSGNTNY	YYCAAPHCNRT----SCHDGFDIWG
Beta-53	CKGSG--HNSPSYWISW	EWMGRIDPSDSYTNY	YYCARHVVALTHL------YPDYWG

Table S4 Cryo-EM data collection, refinement and validation statistics of spike/Fab complexes

Related to Figure 4

* Rigid body refinement only. Numbers in square brackets refer to local refinement results.

	mAb-222 (EMD-13869, PDB 7Q9G)	Beta-6 (EMD-13875, PDB 7Q9P)	Beta-26 (EMD-13871, PDB 7Q9J)	Beta-32 (EMD-13872, PDB 7Q9K)	Beta-43 (EMD-13870, PDB 7Q9I)	Beta-44 (EMD-13874)	Beta-49 (EMD-13857, PDB 7Q6E)	Beta-50 (EMD-13868, PDB 7Q9F)	Beta-53 (EMD-13873, PDB 7Q9M)
Microscope, Detector	eBIC Krios, Falcon-IV Selectris	OPIC Krios, K2 GIF	OPIC Krios, K2 GIF	OPIC Krios, K2 GIF	OPIC Krios, K2 GIF	eBIC Krios, Falcon-IV Selectris	OPIC Krios, K2 GIF	OPIC Krios, K2 GIF	OPIC Krios, K2 GIF
Voltage	300								
Dose(e⁻/Å²)	50.2	40.3	40.3	40.3	40.0	45	38.4	38.4	38.2
Focus range, increment, um	-0.8 to -2.6 (-0.3)								
Nominal magnification kX	165	165	165	165	165	215	165	165	165
Pixel size [super res] (Å)	0.72	0.83	0.82	0.82	0.83	0.576	0.82	0.82	0.82
Symmetry imposed	C1	C1	C1	C1	C1	C1	C1	C1	C1
Particles in final reconstruction	86,742	79,744	50,878	54,370	89,350	61,603	222,689	40,869	32,899
Map resolution (Å) (cryosparc gold-standard FSC 0.143)	3.3	4.5	4.0	4.5	4.9	3.9	2.7	3.6	3.7
Map sharp B factor (Å²)	-83.9	-162.7	-109.5	-150.1	-209.2	N/A	-68.4	-47.6	-36.1
Refinement									
Initial model	7NXA	7PRY	Alphafold VHH. RBD from 7NXA.	RBD from 7NXA, 7PS3.	7Q0I	N/A	7Q0G	7Q0H	7PS2
RBD #up / #fabs	2 / 3	2 / 3	3 / 3	2 / 2	1 (NTD)	2 / 2	0 / 3	0 / 3	2 / 2
Final model:									
Non-hydrogen atoms	28973	30494	30308	27225	30966	N/A	34341	34264	28406
Protein	3632	3807	3797	3388	3881		4327	4312	3613
Ligands	52	52	57	60	53		74	64	43
B factors (Å²)									
Protein	91.1	319.3	202.5	524.5	301.9		65.1	123.5	142.0
Ligand	77.6	267.2	175.9	489.4	283.8		76.2	124.3	124.7
R.m.s. deviations									
Bond lengths(Å)	0.003	0.004	0.004	0.004	0.003	N/A	0.003	0.003	0.004
Bond angles(°)	0.455	0.635	0.529	0.849	0.538		0.479	0.463	0.524
Validation									
Resolution at FSC to model 0.5	3.4	6.4	4.2	8.1	5.4	N/A	2.8	3.7	3.7
CCbox, phenix	0.82	0.81	0.83	0.82	0.76		0.84	0.88	0.84
MolProbity score	1.37	1.91	1.57	1.58	1.47		1.25	1.49	1.39
Clashscore	3.97	8.90	6.17	5.48	4.70		3.20	4.43	4.26
Poor rotamers (%)	0.67	1.20	0.64	0.44	0.80		0.48	0.45	0.75
Ramachandran plot									
Favored (%)	96.9	94.6	96.4	95.9	96.5		97.3	96.1	96.9
Allowed (%)	3.10	4.98	3.56	3.73	3.45		2.70	3.93	2.92
Disallowed (%)	0.00	0.40	0.00	0.36	0.03		0.00	0.00	0.14
